# HAPIFED: a Healthy APproach to weIght management and Food in Eating Disorders: a case series and manual development

**DOI:** 10.1186/s40337-017-0162-2

**Published:** 2017-08-16

**Authors:** Felipe Q. da Luz, Jessica Swinbourne, Amanda Sainsbury, Stephen Touyz, Marly Palavras, Angelica Claudino, Phillipa Hay

**Affiliations:** 1 The University of Sydney, The Boden Institute of Obesity, Nutrition, Exercise & Eating Disorders, Sydney Medical School, Charles Perkins Centre, Camperdown, NSW 2006 Australia; 2 The University of Sydney, School of Psychology, Faculty of Science, Camperdown, NSW 2006 Australia; 3 CAPES Foundation, Ministry of Education of Brazil, Brasília, DF 70040-020 Brazil; 4 Program of Orientation and Attention of Eating Disorders, Federal University of São Paulo, São Paulo, Brazil; 5 Centre for Health Research, School of Medicine, Western Sydney University, Locked Bag 1797, Penrith, NSW 2751 Australia

**Keywords:** Binge eating disorder, Bulimia nervosa, Obesity, Overweight, Integrated treatment

## Abstract

**Background:**

There is a high prevalence of overweight or obesity in people with eating disorders. However, therapies for eating disorders, namely binge eating disorder and bulimia nervosa, do not address weight management. Conversely, weight loss treatments for people with overweight or obesity do not address psychological aspects related to eating disorders. Thus we developed a new treatment for overweight or obesity with comorbid binge eating disorder or bulimia nervosa, entitled HAPIFED (a **H**ealthy **AP**proach to we**I**ght management and **F**ood in **E**ating **D**isorders). This paper describes HAPIFED and reports a case series examining its feasibility and acceptability.

**Methods:**

Eleven participants with overweight or obesity and binge eating disorder or bulimia nervosa were treated with HAPIFED in two separate groups (with once or twice weekly meetings). Weight, body mass index (BMI) and eating disorder symptoms, as well as depression, anxiety and stress, were assessed at baseline and at the end of the 20-session HAPIFED intervention.

**Results:**

Eight of the 11 participants completed the intervention, with diverse results. Six of the 8 participants who completed HAPIFED reduced their weight between baseline and the end of the intervention. Median scores on the Eating Disorder Examination Questionnaire for binge eating, restraint, and concerns about eating or weight and shape, were reduced in the group overall between baseline and the end of the intervention. One participant, who at baseline was inducing vomiting and misusing laxatives in an attempt to lose weight, reduced these behaviors by the end of the intervention. Three participants at baseline were undertaking episodes of compulsive exercise, and they reduced or stopped this behavior, but one participant commenced episodes of compulsive exercise by the end of the intervention. All participants who completed the intervention rated the suitability and success of HAPIFED as 7 or more out of 10 (0 = not at all suitable/successful; 10 = extremely suitable/successful).

**Conclusion:**

This case series supports the feasibility and acceptability of HAPIFED as a potential new treatment for overweight or obesity with comorbid binge eating disorder or bulimia nervosa. Clinical trials are necessary to examine the efficacy and effectiveness of HAPIFED.

**Trial registration:**

Australian and New Zealand Clinical Trials Registry (Universal Trial Number): U1111–1149-7766. Date of registration: 4th November 2013.

**Electronic supplementary material:**

The online version of this article (doi:10.1186/s40337-017-0162-2) contains supplementary material, which is available to authorized users.

## Plain English Summary

Obesity is a growing problem, and its co-morbidity with disorders of binge eating is growing faster than either problem on its own. Persistent binge eating helps explain why usual ‘diets’ often fail. We have developed a new approach that integrates the best of current behavioral weight loss therapies with the leading psychological therapy for disorders of binge eating, namely cognitive behavior therapy. This new approach, named HAPIFED (a **H**ealthy **AP**proach to we**I**ght management and **F**ood in **E**ating **D**isorders), aims to help people to achieve sustained weight loss by reducing disordered eating and enhancing psychological wellbeing. This paper presents the outcome in a small number of people treated with this new approach. We also include a treatment manual (Additional file 1) that was refined after our initial experience with this new approach. The treatment was acceptable to the participants and appeared helpful in reducing weight and binge eating. However, there was no control group. Larger studies with a control group are needed to test this new approach.

## Background

The occurrence of binge eating disorder (BED) or bulimia nervosa (BN) in individuals with overweight or obesity can range from 51.6% amongst people enrolling for weight loss programs [1] to 87.8% in clinic surveys [2]. Moreover, recent epidemiological evidence shows that while the independent prevalence of eating disorder behaviors – notably binge eating and very strict dieting/fasting – and obesity have increased steadily in the past 20 years, there were even higher increases in the prevalence of eating disorder behaviors with comorbid obesity [3]. There is good evidence supporting the use of psychological therapies for the treatment of BED [4–6] and BN [4, 7, 8], however these therapies are not designed to assist with weight loss in people with comorbid overweight or obesity. On the flip side, behavioral weight loss therapies (BWLTs) can reduce body weight and binge eating in individuals with obesity. One example of a BWLT is the LEARN program (Lifestyle Exercise Attitudes Relationships Nutrition) [9], which effectively reduced both excess weight and binge eating in participants of a clinical trial involving 16 sessions over a 24-week period [10]. Nonetheless, there are three important limitations with BWLTs. Firstly, weight regain after BWLTs is common [11], as is true of any weight loss intervention [12, 13]. A second limitation is that BWLTs were not designed to treat eating disorders psychopathology. A third limitation is that to our knowledge, BWLTs have not been studied in people with BN, however this is necessary due to the increase in the prevalence of overweight and obesity in patients with BN [14]. The development of interventions that result in sustainable reductions in excess body weight while also treating BED and BN would be of benefit, because individuals with overweight or obesity and comorbid eating disorders have been shown to benefit – in terms of weight management and reduction of binge eating – as a result of diet-induced weight loss [15–17]. Experts in the field usually recommend treating the eating disorder first, and attempts to reduce weight are recommended only once the eating behavior has stabilized [4]. However, an intervention simultaneously targeting eating disorders and weight loss would likely be appealing to the target population, because individuals with obesity and co-morbid eating disorders seek weight loss treatments in preference to treatments for disordered eating [18–22]. Furthermore, continued binge eating may contribute to the high relapse rates observed in weight loss treatments [23]. Thus, in this work we developed and tested an intervention that addresses the limitations of existing approaches for individuals with overweight or obesity and co-morbid BED or BN.

The specific aim of this research was to develop and pilot test a new multi-disciplinary mutualized therapy that integrates a BWLT for obesity [24, 25] with the leading psychotherapeutic intervention for eating disorders (cognitive behavior therapy – enhanced, or CBT-E) [4]. We named this new therapy HAPIFED (a **H**ealthy **AP**proach to we**I**ght management and **F**ood in **E**ating **D**isorders). This paper details the HAPIFED manual and describes a case series of individuals with overweight or obesity and comorbid eating disorders (BED or BN) who completed a pilot HAPIFED intervention. The feasibility and acceptability of HAPIFED were specifically examined.

## Methods

### Description of the treatment

HAPIFED is an extension of CBT-E for eating disorders [4], via integration into the program of BWLT [24, 25] and multidisciplinary education about nutrition and exercise (see Table 1). HAPIFED departs from the focus on avoiding or reversing restrictive dieting that is part of CBT-E, and instead emphasizes eating according to bodily hunger and satiety signals [24, 25], with the aim of promoting moderate weight loss. HAPIFED also promotes active behavior change through behavioral experiments that participants engage in between sessions.

**Table 1:** Comparison of characteristics of CBT-E, BWLT and HAPIFED

Treatment components Included	CBT-E [4]	BWLT [9]	HAPIFED [4]
Personalised eating disorder CBT formulation	Yes, for eating disorder only	No	#Yes, for eating disorder and overweight/obesity
Psycho-education	Yes, for eating disorder only	Yes, for overweight/obesity only	Yes, for eating disorder and overweight/obesity
Nutritional counselling	Yes, not dietician led	Yes, dietician led	Yes, dietician led
Behavioural monitoring	Yes	Yes	Yes (including appetite cues)
Socratic questioning (cognitive therapy)	Yes	No	Yes
Multidisciplinary	No	Yes	Yes
Session (number)	20	16	20*
Weight loss management included	No	Yes	Yes
Behavioural activation	No	Yes	Yes
“Healthy” exercise promoted	No	Yes	Yes
Emotion regulation skills	Yes	No	Yes

# This includes the role of dietary restriction in contributing to binge eating. *In the modifications made to HAPIFED subsequent to this study, the session number has been increased to 30. This table was adapted from Palavras et al. *Trials*. 2015 [41]. CBT-E = Cognitive behaviour therapy – enhanced; BWLT = Behavioral Weight Loss Therapy; HAPIFED = a **H**ealthy **AP**proach to we**I**ght management and **F**ood in **E**ating **D**isorders

In the current study, all HAPIFED sessions were group-based and were of 90-min’ duration. The HAPIFED program involved a total of 20 group sessions with a Doctoral-level clinical psychologist and a Masters-level co-therapist that were trained and supervised to conduct HAPIFED. In addition to the standard CBT-E protocol [4], HAPIFED included conjoint sessions for nutritional counseling (one session lead by a dietitian), healthy exercise (one session lead by an exercise physiologist) and BWLT (two sessions). HAPIFED incorporated approaches that included healthy lifestyle changes and appetite awareness, with the aim of reducing eating as a means of regulating emotions. Approaches were informed by an understanding of the effects of starvation [24]. Monitoring and encouragement of food intake according to levels of physical hunger and satiety, consumption of vegetables and fruits, and moderate exercise continued throughout HAPIFED. Each stage of HAPIFED is described in greater detail below and summarized in Table 2, and the current version of the full manual is appended (Additional file 1).

**Table 2:** Brief summary of the main activities/goals of each session of the HAPIFED pilot study

STAGE 1	
Session 1	Information about the treatment: real-time recording of eating behavior, weighing and treatment goals. Psychoeducation on eating disorders and the transdiagnostic cognitive-behavioral model of eating disorders.
Session 2	Assessment of participants’ real-time recording of their eating behavior and assessment of their eating habits. Psychoeducation on eating disorders continues. Information on behavioral weight loss therapy.
Session 3	Psychoeducation on the importance of regular eating. Information on physical activity including discussion on healthy and excessive exercise.
Session 4	Review of participants’ understanding of the behavioral weight loss therapy. Discussion on purging and its negative consequences. Nutritional education.
Session 5	Discussion of factors that can lead to and maintain binge eating. Discussion on how feelings of fullness after eating can trigger eating disorder behaviors.
Session 6	Discussion of participants’ engagement in physical activity. Review of participants’ understanding of the difference between healthy and excessive exercising.
Session 7	Discussions on how significant others (e.g. family and friends) can be helpful in helping participants improve their eating behaviors. Mindful eating activity and discussion.
STAGE 2	
Session 8	Slow breathing activity. Review of participants’ progress with the treatment and identification of potential barriers to change. Review and personalization of the transdiagnostic cognitive-behavioral model of eating disorders.
Session 9	Progressive muscle relaxation. Review of participants’ progress with the treatment and identification of potential barriers to change. Review and personalization of the transdiagnostic cognitive-behavioral model of eating disorders.
STAGE 3	
Session 10	Discussion on the negative consequences of overvaluation of the importance of body shape and weight. Activity on helping participants identify parts of their bodies that they like.
Session 11	Discussion on the importance of other domains of self-evaluation not related to body shape or weight. Activity on identifying participants’ positive characteristics (not related to body shape or weight).
Session 12	Discussion on the importance of participants engaging in self-nurturing activities in order to maintain a positive mindset and how this can be beneficial for their eating behavior. Discussion on excessive body shape checking or avoidance, and their negative consequences.
Session 13	Discussion on factors that can contribute to participants’ “feeling fat”. Discussion on ways of increasing self-confidence and its benefits.
Session 14	Therapists focus on Socratic questioning in order to help participants identify their cognitive distortions regarding “feeling fat” and other dysfunctional thoughts related to their eating disorder. Review and discussion of the transdiagnostic cognitive-behavioral model of eating disorders.
Session 15	Discussion on black/white (dichotomous) thinking and perfectionism related to eating disorders. Activity on helping participants reflect on the origins of overvaluation of the importance of body shape and weight.
Session 16	Discussion on the “eating disorders mindset”. Discussion on how participants could create and maintain social connections with people that are supportive (and not detrimental) of their recovery from the eating disorder.
Session 17	Discussion on the negative effects of strict and unhealthy dietary restraint. Discussion on the negative consequences of overvaluation of the importance of body shape and weight, and the benefits of developing other core values and engagement in healthy activities related to these new values.
STAGE 4	
Session 18	Discussion of relapse prevention strategies. Discussion on strategies to respond in a healthy manner to event-related negative changes in eating. Activity on proactive problem solving.
Session 19	Discussion on residual binges by reviewing factors that may contribute to their maintenance. Discussion on strategies to respond in a healthy manner to mood-related negative changes in eating. Discussion on unhelpful thinking styles that contribute to the maintenance of eating disorders.
Session 20	Discussion on participants’ plans for healthy eating after the treatment has finished, relapse prevention and new core values. End of treatment.

### Stages of treatment

In Stage 1 (Sessions 1–7), participants were provided with information about eating disorders, an introduction to the transdiagnostic cognitive-behavioral model of eating disorders [4], and information on non-hungry and hungry eating. In-session weighing and monitoring of eating behavior were also commenced. Monitoring forms were provided to participants, and therapists asked participants to take note – in real-time – of food/drinks consumed, their level of hunger and satiety before and after eating/drinking, the place where they ate/drank, any eating disorder behaviors (e.g. binge eating, inducing vomiting etc.), as well as any situation that could have influenced their eating behavior. In this stage, a session outlining hunger regulation as outlined previously [24] was conducted. Specific steps were outlined to inform patients on eating according to their hunger and satiety signals in order to promote weight loss. Additionally, participants were encouraged to eat 5 or more serves of vegetables and 2 or more serves of fruit per day, and to gradually build up to a level of physical activity that is considered necessary for the prevention of weight regain after weight loss (up to 10,000–12,000 steps per day or its equivalent in other activities) [26]. Stage 1 also included information sessions on general nutrition and exercise. Exercise was addressed using the Loughborough Eating Disorders Activity Program (LEAP) [27], with the goal of incorporating “healthy” exercise, instead of insufficient or compulsive exercise that is often seen in individuals with weight and eating disorders [28]. All in all, these instructions encouraged healthy lifestyle changes that are known to bring about modest weight loss [29, 30]. Therapists also discussed with participants how their relatives and friends could potentially support them with establishing and maintaining healthy eating habits. Lastly, in Stage 1, an activity on mindful eating was conducted with participants.

Stage 2 (Sessions 8–9) included the practice of relaxation activities (namely slow breathing and progressive muscle relaxation), followed by a review of participants’ progress with the treatment. Stage 2 also included a personalization of the transdiagnostic cognitive-behavioral model of eating disorders [4]. During these sessions, participants were instructed to revisit aspects of the model, such as over-evaluation of weight and shape and their control, strict dieting, as well as events and associated mood change, and the influence of these parameters on their eating behaviors. Participants’ monitoring of their eating behaviors (as described in Stage 1) was revised regularly. Participants were also reminded of the steps that were described in Stage 1 and that are helpful in promoting weight loss.

In Stage 3 (Sessions 10–17), monitoring of eating and weight control behaviors (including eating according to physical hunger and satiety signals, or binge eating, excessive body checking or avoidance, exercise, and the urge to compulsively over-exercise or engage in other compensatory behaviors in response to eating, such as vomiting) continued, with an additional emphasis on behavioral experiments. The behavioral experiments occurred by asking participants to engage between sessions in individualized therapeutic activities that help to oppose eating disorder behaviors (e.g. suggesting to a participant to engage in some alternatively pleasurable activity such as going for a walk in the park when feeling tempted to binge eat). Behavioral experiments were introduced in order to decrease eating disorder symptoms, establish regular meal patterns, and promote self-control. Other components that were introduced in Stage 3 were activities that helped participants to develop awareness of parts of their bodies and their personal characteristics that they like, self-nurturing activities, as well as activities to increase participants’ confidence. Challenging the beliefs and attitudes that tend to reinforce eating disorder behaviors, such as valuing oneself according to one’s weight and shape, and “all or nothing” (black/white, or dichotomous) thinking, were employed. The origins and negative consequences of self-evaluation that is excessively focused on weight, shape or eating, were discussed.

Stage 4 (Sessions 18–20) involved relapse prevention strategies specially focused on residual binge eating episodes triggered by adverse events and mood. Proactive problem-solving was also incorporated at this stage, in order to help participants deal with situations that trigger eating disorder behaviors. During this last stage, there was continuing reinforcement of participants’ monitoring of their eating behaviors as well as other aspects of the BWLT (consumption of 5 or more serves of vegetables and 2 or more serves of fruit per day, and aiming to undertake physical activities to the equivalent of 10,000–12,000 steps per day).

### Participants

Participants were recruited through online announcements in the staff news at the University of Sydney and via advertisement in a local newspaper (mX News Media, Surry Hills, New South Wales, Australia). Inclusion criteria were body mass index (BMI) > 27 kg/m^2^, age > 18 years, and primary diagnosis of BED or BN according to the criteria in the Diagnostic and Statistical Manual of Mental Disorders, fifth edition (DSM-5) [31]. Exclusion criteria were diagnosis of psychosis, bipolar disorder or a high level of suicide risk. The Mini International Neuropsychiatric Interview (MINI) [32] was used to assess eating disorders and other mental health features related to the inclusion and exclusion criteria. Assessments were performed by a research assistant who also assessed other exclusion criteria, namely current use of weight loss medication, a history of bariatric surgery, or medical conditions (e.g. Prader-Willi syndrome, Cushing’s syndrome) or use of medications (e.g. insulin, hydrocortisone) that interfere with appetite control. Those who met eligibility criteria were asked to provide written informed consent prior to participation in the trial.

**Fig. 1:**
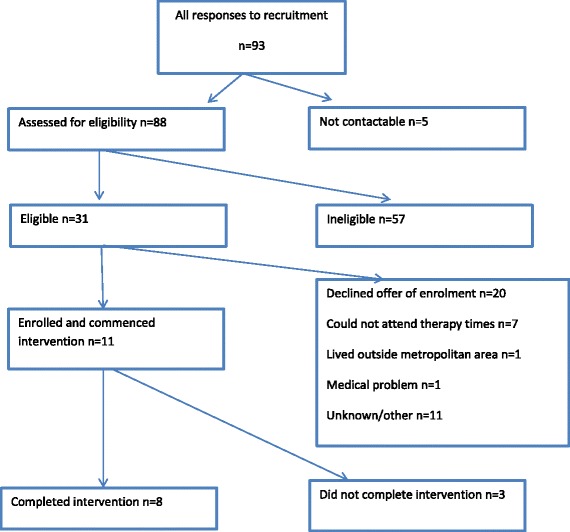
Participant flow

Post-treatment assessment occurred at the end of the last treatment session (session 20). There were 11 participants commencing the treatment (see Fig. 1): six were treated in a group with twice-weekly sessions, and five were treated in a group with once weekly sessions. Of these, five out of six and three out of five, respectively, attended Session 20 of their respective groups. Of the 11 participants who commenced treatment, 3 withdrew after the first session, with 2 of these participants citing conflicting work schedules and 1 indicating that the program was not suitable for them. Of the 8 participants that attended Session 20 (the last treatment session), 7 participants attended 14 or more sessions (70% attendance), and 1 participant attended 12 out of 20 sessions (60% attendance). All of these 8 participants were female, aged from 28 to 69 years, with BMI from 28 to 36.8 kg/m^2^ at treatment commencement. Six of these 8 participants were employed and 2 were unemployed. In addition to the diagnosis of BED or BN, some participants also met criteria for other mental health disorders, namely major depressive disorder (3 participants), posttraumatic stress disorder (2 participants), social phobia (2 participants), obsessive compulsive disorder (1 participant) and generalized anxiety disorder (1participant) (see Table 3).

**Table 3:** Participants’ psychiatric diagnosis, weight, BMI and eating disorder symptoms at baseline and at the end of the HAPIFED treatment

Participant ID	ED Diagnosis and comorbidities	Weight (kg)		Weight change (%)	BMI Kg/m^2^		EDE-Q																			
							Restraint		Eating concerns		Weight concerns		Shape concerns		Global score		OBE		SBE		Vomit		Laxatives		Compulsive exercise	
		Pre	Post		Pre	Post	Pre	Post	Pre	Post	Pre	Post	Pre	Post	Pre	Post	Pre	Post	Pre	Post	Pre	Post	Pre	Post	Pre	Post
1	BN MDD SP OCD	110.9	111.7	+0.7%	40.7	41.0	1.4	2.2	4.6	4.4	5.4	5.2	5.7	5.4	4.2	4.3	12	6	4	5	0	0	0	0	0	0
2	BED	88.1	87.1	- 1.1%	33.5	33.1	2.0	2.6	5.2	1.6	4.6	4.6	4.9	4.0	4.2	3.2	10	10	10	08	0	0	0	0	0	0
3	BED MDD	85.1	85.8	+0.8%	32.8	33.1	3.6	2.4	1.6	0.8	2.8	1.6	3.3	2.0	2.8	1.7	4	14	0	0	0	0	0	0	0	0
4	BN	82.6	81.6	- 1.2%	28.5	28.2	3.2	0.6	4.4	1.0	4.2	2.4	5.1	3.5	4.2	1.8	18	2	8	4	20	3	28	3	4	0
5	BN	85.8	84.0	- 2.0%	31.9	31.2	1.8	1.8	3.4	2.4	5.6	3.2	5.8	4.3	4.1	2.9	5	3	10	0	0	0	0	0	15	3
6	BN MDD PTSD	106.4	104.3	- 1.9%	36.8	36.0	0.6	3.2	3.0	2.6	3.2	3.2	4.2	4.3	2.7	3.3	4	12	0	10	0	0	0	0	0	5
7	BED	106.3	105.1	- 1.1%	39.0	38.6	2.4	1.2	5.6	1.4	5.2	1.4	6.0	3.3	4.8	1.8	25	5	0	5	0	0	0	0	0	0
8	BN PTSD SP GAD	79.8	75.0	- 6%	33.2	31.2	6.0	3.6	4.2	0	4.4	2.4	5.7	2.2	5.0	2.0	15	0	10	0	0	0	0	0	15	0
Median change (IQ range)		−1.1 (−1.9 to −0.1)			−0.4 (−0.75 to 0)		−0.6 (−1.7 to 0.7)		−2.4 (−3.9 to −0.6)		−1.5 (−2.2 to −0.1)		−1.4 (−2.1 to −0.6)		−1.1 (−2.7 to −0.4)		−4.0 (−15.5 to 4.0)		−1.0 (−7.0 to 3.0)		0 (0 to 0)		0 (0 to 0)		0 (−8.0 to 0)	

*HAPIFED* a **H**ealthy **AP**proach to we**I**ght management and **F**ood in **E**ating **D**isorders, *ID* identification number, *ED* Eating disorder, *BMI* Body mass index, *EDE-Q* Eating Disorder Examination Questionnaire, *Pre* pre-treatment (weight at Session 1), *Post* post-treatment (weight at Session 20), *BN* Bulimia nervosa, *MDD* Major depressive disorder, *SP* Social phobia, *OCD* Obsessive-compulsive disorder, *BED* Binge eating disorder, *PTSD* Posttraumatic stress disorder, *GAD* Generalized anxiety disorder, *IQ* Interquartile, *OBE* Objective binge eating, *SBE* Subjective binge eating

### Measures

The following measures were made at baseline and at the end of the treatment, unless stated otherwise.

#### Physical measures

Participants were weighed without shoes or any heavy clothes (e.g. jackets) before treatment and once a week throughout treatment, at the group session. Participants who attended the group with sessions twice a week were also weighed only once a week. As all sessions were held in the morning, participants were always weighed in the morning. Participants’ height was measured only at the baseline assessment using a stadiometer, and this was used to calculate BMI before and after treatment.

#### Eating Disorder Examination Questionnaire (EDE-Q)

The EDE-Q is the self-report questionnaire based on the “gold standard” Eating Disorder Examination (EDE) for the assessment of eating disorder symptoms [33, 34].

#### Depression Anxiety and Stress Scale (DASS-21)

The 21-item DASS [35–37] is a self-report questionnaire that was used to assess symptoms of depression, anxiety and stress.

#### Mini International Neuropsychiatric Interview (MINI)

The MINI [32] is a structured diagnostic interview designed to assess the existence of mental disorders. It was used at baseline only, to assess participants’ eating disorders and comorbidity with other psychiatric disorders. The MINI was conducted by a research assistant with each participant individually. This interview lasts for approximately 15 min and provides an accurate assessment of mental disorders as defined in the DSM-5 [31].

#### Participants’ acceptance of the treatment

In order to examine the acceptability of the treatment, at the end of the program participants were asked to give their opinion, as a rating, on the suitability and success of the treatment. This took the form of a short questionnaire with the following two questions: How suitable do you think the treatment was for your problem? How successful do you think the treatment here has been? Participants’ answers could range from 0 (not at all suitable/successful) to 10 (extremely suitable/successful). This rating occurred blinded to the therapists with participants rating this in a short questionnaire administered by a research assistant.

#### Statistical analysis

In order to have a quantitative analysis of the data, we chose to calculate medians and interquartile ranges rather than means and standard deviations, because means and standard deviations are subject to biasing when using small sample sizes, such as the small number of participants assessed in this study. Additionally, we reported only descriptive results and did not compute *p*-values.

## Results

### Weight

Participants’ weight change from baseline to the end of the treatment included six participants that lost weight and two participants that gained weight (Table 3).

### Eating disorder symptoms

Scores on the EDE-Q (Table 3) showed the following changes in eating disorder symptoms. Changes in restraint from baseline to end of treatment were diverse (four participants reduced restraint, one showed no change and three increased restraint). All of the eight participants reduced concerns about eating by the end of the treatment, in comparison to their baseline measures. Six and seven out of eight participants reduced concerns about weight and shape, respectively, at the end of the treatment in comparison to their baseline measures, and one participant increased concern about shape (albeit only by 0.1 points) by the end of the treatment in comparison to baseline measurement. Six participants reduced their global score on the EDE-Q, and two participants showed slightly increased scores (from 4.2 to 4.3 and from 2.7 to 3.3).

Diverse changes in frequency of binge eating episodes occurred from baseline to the end of the treatment. In regards to objective binge eating episodes (OBE), five participants showed reduced, one showed no change and two showed increased episodes at the end of treatment. In regards to subjective binge eating episodes (SBE), four showed reduced, one showed no change and three showed increased episodes from pre-treatment (baseline) to post-treatment.

Five participants met criteria for BN at baseline, however their compensatory behaviors were diverse. One participant reported inducing vomiting and using laxatives, two participants reported episodes of driven or compulsive exercise and two participants reported fasting as a means of trying to control their weight or shape. The following changes occurred in regards to compensatory behaviors (namely vomiting, use of laxatives and driven or compulsive exercise). The only participant who reported vomiting and using laxatives as a means of trying to control weight or shape reduced these behaviors from baseline to the end of the treatment. Three participants reported episodes of driven or compulsive exercise as a means of controlling weight or shape at baseline, and reduced this behavior by the end of the treatment. One participant showed onset of episodes of driven or compulsive exercise at the end of treatment. It is noteworthy that during the program, some participants appeared to have difficulty understanding the difference between healthy and driven or compulsive exercise, and this may have influenced the increase in driven or compulsive exercise seen in the participant mentioned above.

### Symptoms of depression, anxiety and stress

The changes in scores of depression, anxiety and stress from baseline to the end of treatment are shown in Table 4. For symptoms of depression, six participants showed a reduction and two showed an increase from pre- to post-treatment assessments. In regards to symptoms of anxiety, five participants showed a reduction, one had no change and two showed increased symptoms from start to end of treatment. In regards to symptoms of stress, five showed reduced and three showed increased symptoms over the course of treatment.

**Table 4:** Participants’ symptoms of depression, anxiety and stress at baseline and in the end of the HAPIFED treatment, as well as participants’ opinion about how suitable and successful the treatment was

Participant ID	DASS-21						Suitable treatment^a^	Successful treatment^a^
	Depression		Anxiety		Stress			
	Pre	Post	Pre	Post	Pre	Post		
1	24	20	10	14	14	18	9	8
2	22	14	16	12	20	10	7	7
3	2	10	0	0	0	6	10	8
4	16	4	2	0	12	4	10	9
5	8	0	12	4	26	4	10	8
6	28	22	16	18	24	26	7	8
7	18	14	8	4	22	16	8	8
8	38	42	18	12	28	26	10	10
Median change (IQ range)	−5.0 (−8.0 to 0)		−3.0 (−5.0 to 1.0)		−4.0 (−9.0 to 3.0)		8.5 (7.0 to 10.0)	8.0 (8.0 to 8.5)

*HAPIFED* a **H**ealthy **AP**proach to we**I**ght management and **F**ood in **E**ating **D**isorders, *ID* identification number, *DASS-21* Depression, Anxiety and Stress Scale (21-item), *Pre* pre-treatment (baseline), *Post* post-treatment (end of treatment), *IQ* Interquartile. ^a^ Scores ranged from 0 (not at all suitable/successful) to 10 (extremely suitable/successful)

### Participants’ acceptance of the treatment

All participants scored equal to or higher than seven out of ten in regards to how suitable and successful they rated the treatment (see Table 4).

## Discussion

The aim of this study was to examine the feasibility and acceptability of a new treatment for eating disorders (BED or BN) with comorbid overweight or obesity, named HAPIFED, using a case series. Eight participants were assessed at baseline and at the end of treatment in regards to weight status, eating disorder symptoms, mental health status (symptoms of depression, anxiety and stress) and their perception of suitability and success of the treatment.

Overall the changes in regards to weight and eating disorder symptoms were positive. Six out of eight participants exhibited a reduction in weight and six out of eight participants showed a reduction in eating disorder symptoms (according to global EDE-Q scores) at the end of the intervention in comparison to their baseline measures. Some individuals showed increases in weight or eating disorder symptoms, namely restraint, binge eating, and compulsive exercise. In regards to the increase in restraint, it is noteworthy that while this is usually considered a symptom of eating disorders [4], for some individuals with overweight or obesity, a reasonable increase in restraint is actually positive and beneficial in assisting with weight loss [38]. During this pilot study we observed that some participants struggled to engage in healthy levels of exercise, as indicated by their engaging only in the opposite extremes of sedentary behavior or excessive exercise.

The results of this pilot study enabled further development of the therapy in order to potentially optimize it for individuals with BED or BN with comorbid overweight or obesity. Modifications to the therapy implemented in this pilot study were made and a new HAPIFED manual (Additional file 1) was developed. Modifications included the addition of a bi-weekly review of participants’ progress with eating according to physical hunger and satiety signals, consumption of adequate quantities of vegetables and fruits, as well as engagement in regular physical activity, in order to promote weight loss. We also recommend in the new HAPIFED manual that in future use of the program, pedometers be provided to participants in order to facilitate monitoring and motivation for physical activity, with a clearer emphasis on healthy versus excessive or driven exercise. Moreover, 20 sessions were considered too few for effective treatment, and 30 sessions are recommended in the revised HAPIFED manual, because extended care in the context of recovery from binge eating disorders was found to predict better outcomes [39]. Participants found it onerous to attend HAPIFED sessions twice weekly during work hours. As such, once weekly and/or after work hours would likely have enabled more people to attend regularly. Additional changes to the revised HAPIFED manual involved increases in the number of nutritional education sessions and physical activity educations sessions, development of a formulation of disordered eating and weight gain, increased focus on problem solving, training in interpersonal skills, brainstorming with participants on helpful modifications to the home environment, restructuring of cognitive distortions, and an increased number of sessions on relapse prevention.

This study had some limitations. The number of cases presented is small and is thus not representative of patients with BED or BN and comorbid overweight or obesity. Participants were submitted to treatments with different frequency (once or twice a week), and this may have influenced the outcomes. Four out of eight cases presented met criteria for other comorbid psychiatric diagnoses in addition to BED or BN, and this may have influenced their response to treatment. Furthermore, this study does not provide information regarding causal effects of the treatment on weight, eating disorder symptoms or mental health due to its methodological design, not being a randomized controlled trial. Finally, symptoms were assessed with the self-report EDE-Q and not with the structured interview, namely the Eating Disorder Examination (EDE) [4]. Therefore, future randomized controlled trials to test the efficacy of HAPIFED in comparison to a control intervention are needed, along with effectiveness trials to test the effects of this new treatment in real world clinical practices. Future research examining the treatment of impulsivity in individuals with binge eating disorders [40] and comorbid overweight or obesity may also improve the understanding of additional treatment components to reduce binge eating.

## Conclusion

In summary, changes in weight, eating disorder symptoms, mental health and acceptability of treatment were examined in a case series of participants with BED or BN with comorbid overweight or obesity treated with HAPIFED. This research showed that most participants in the case series had lower weight, reduced eating disorder symptoms and improved mental health status at the end of treatment in comparison to baseline. At the end of the intervention, all participants reported that HAPIFED was a suitable and successful treatment. This study supports the feasibility and acceptability of treating BED or BN with comorbid overweight or obesity. This study also enabled the development of an improved HAPIFED manual that requires evaluation in future clinical trials.

## Additional file

Additional file 1: HAPIFED: A Healthy APproach to weIght management and Food in Eating Disorders. 30 Group Session Program Manual©.(PDF 1410 kb)

## Abbreviations

BED: Binge eating disorder
BN: Bulimia nervosa
BWLT: Behavioral weight loss therapy
CAPES: Coordenação de aperfeiçoamento de pessoal de nível superior
CBT-E: Cognitive behavior therapy – enhanced
DASS-21: Depression anxiety and stress scale
DSM-5: Diagnostic and statistical manual of mental disorders, fifth edition
EDE: Eating disorder examination
EDE-Q: Eating disorder examination questionnaire
HAPIFED: Healthy APproach to weIght management and Food in Eating Disorders
LEAP: Loughborough eating disorders activity program
LEARN: Lifestyle exercise attitudes relationships nutrition
MINI: Mini international neuropsychiatric interview
NHMRC: National health and medical research council
OBE: Objective binge eating
SBE: Subjective binge eating
