# Supplementary material for: HAPIFED: a Healthy APproach to weIght management and Food in Eating Disorders: a case series and manual development
Source: J Eat Disord. 2017 Aug 16;5:29. doi: 10.1186/s40337-017-0162-2 (PMC5558732; doi:10.1186/s40337-017-0162-2)
Supplement: Additional file 1: — HAPIFED: A Healthy APproach to weIght management and Food in Eating Disorders. 30 Group Session Program Manual©.(PDF 1410 kb) [file 40337_2017_162_MOESM1_ESM.pdf]

HAPIFED: A Healthy APproach to weIght management and Food in Eating Disorders

30 Group Session Program Manual ©

Felipe Q da Luz, Jessica Swinbourne, Phillipa Hay, Stephen Touyz, Marly Palavras, Angelica Claudino, Amanda Sainsbury

HAPIFED is a manualized program for individuals with comorbid binge eating disorder or bulimia nervosa and overweight or obesity (body mass index $\geq$ 25 kg/m^2). This program combines the evidence-based standard therapy for eating disorders, which is cognitive behavioral therapy – enhanced for eating disorders (CBT-E) (1) (excluding interventions for underweight individuals) with behavioral weight loss therapy (2, 3) and multidisciplinary strategies to promote moderate weight loss.

HAPIFED has been developed for people with binge eating disorder or bulimia nervosa and overweight / obesity who are seeking treatment for weight loss. It should be noted that HAPIFED is not suitable for individuals who are at normal weight, who are underweight or who are not seeking weight loss therapy.

In order to obtain a thorough understanding of HAPIFED, the following references are recommended: *Cognitive behavior therapy and eating disorders* (1) by Christopher Fairburn; *Eating disorders: advances in psychotherapy – evidence-based practice* (4) by Stephen Touyz, Janet Polivy and Phillipa Hay; *The don't go hungry diet* (2) and *Don't go hungry for life (3)*, by Amanda Sainsbury-Salis; *The LEARN program for weight management* (5) by Kelly Brownell, and the accompanying paper reporting the HAPIFED pilot study *HAPIFED: a Healthy APproach to weIght management and Food in Eating Disorders: a case series and manual development* by Felipe Q. da Luz *et al*.

It is recommended to run the HAPIFED Program in a group setting with 6 to 8 participants, with weekly frequency and implemented by one therapist, with or without the support of an additional co-therapist.

Table of contents

Program overview 1
List of abbreviations 2
Individual assessment prior to program commencement 3
Stage 1: Introduction and psychoeducation 5

Session 1: Introduction 6
Session 2: Psychoeducation about weight and behavioral weight loss 20
Session 3: Psychoeducation about eating disorders 23
Session 4: Nutritional education and problem solving 29
Session 5: Physical activity education 33
Session 6: Healthy versus excessive exercise 42
Session 7: Nutritional education (continued) 44

Stage 2: Core interventions 46

Session 8: Changes in eating related to events and moods 47
Session 9: Changes in eating related to events and moods (continued) 50
Session 10: Mindfulness / Social environment 53
Session 11: Dissatisfaction with body weight and shape 57
Session 12: Dissatisfaction with body weight and shape (continued) 61
Session 13: Body checking, comparison and avoidance 65
Session 14: Physical activity education (continued) 68
Session 15: Nutritional education 69
Session 16: Increasing self-confidence 71
Session 17: Relaxation / Barriers to change 74
Session 18: Progressive muscle relaxation / Barriers to change (continued) 77
Session 19: Personalization of the formulation 80
Session 20: Unhelpful thinking styles 84
Session 21: Unhelpful thinking styles (continued) 92
Session 22: Getting social support 94
Session 23: Positive self-evaluation 96
Session 24: Positive self-evaluation (continued) 99
Session 25: Nutritional education (final review) 101
Session 26: Open session 103

Stage 3: Relapse prevention 104

Session 27: Relapse prevention (Part 1) 105
Session 28: Relapse prevention (Part 2) 108
Session 29: Relapse prevention (Part 3) 109
Session 30: Relapse prevention (Part 4) 113

List of handouts 115
References 116

Program overview

The HAPIFED Program is divided into the following three stages and activities:

Stage 1 (Sessions 1 to 7): Introduction and psychoeducation

- Engage the participant in the program and in the process of change.
- Provide relevant psychoeducation about obesity, weight loss and eating disorders.
- Provide initial education about nutrition and physical activity.
- Introduce proactive problem-solving techniques.
- Explain the difference between healthy versus disordered eating and exercise habits.

Stage 2 (Session 8 to 26): Core interventions

- Address changes in eating related to events and mood.
- Encourage participants to improve their interpersonal functioning and fortify their supportive relationships.
- Guide participants through mindfulness and relaxation exercises.
- Address dissatisfaction with body image and weight.
- Address unhealthy body checking, comparison and avoidance.
- Encourage participants to engage in self-nurturing activities.
- Improve participants' knowledge regarding nutrition and physical activity, and motivate participants to continue engagement with behavioral weight loss strategies.
- Help participants to identify any existing or emerging barriers to change.
- Help participants to personalize their HAPIFED formulation and adjust interventions according to their individualized formulation.
- Use cognitive therapy and behavioral experiments in order to help participants to restructure any unhelpful thinking styles they may have and reduce their disordered eating behaviors.
- Encourage participants to engage in activities that enhance positive self-evaluation and are not related to shape, weight or eating.

Stage 3 (Sessions 27 to 30): Relapse prevention

- Help participants to think about strategies to maintain healthy eating and healthy physical activity levels after program completion.
- Encourage the maintenance of any improvements in eating disorder symptoms.
- Help participants to develop a detailed post-program management plan.

30 Group Session Program Manual © da Luz, Swinbourne, Hay, Touyz, Palavras, Claudino, Sainsbury 2017

List of abbreviations

BED	Binge eating disorder
BMI	Body mass index
BN	Bulimia nervosa
CBT-E	Cognitive behavior therapy – enhanced
CD-Quest	Cognitive distortions questionnaire
DSM-5	Diagnostic and statistical manual of mental disorders – fifth edition
GP	General practitioner
HAPIFED	A healthy approach to weight management and food in eating disorders
LEARN	Lifestyle exercise attitudes relationships nutrition
PhD	Doctor of philosophy
OSFED	Other specified feeding or eating disorder
UFED	Unspecified feeding or eating disorder

Individual assessment prior to program commencement

This is an individual session between the therapist and a potential participant. In this session, the therapist's aims are the following:

To determine whether the potential participant meets the following eligibility criteria

- Has a body mass index $\geq$ 25 kg/m^2
- Meets diagnostic criteria for binge eating disorder (BED), bulimia nervosa (BN), other specified feeding and eating disorder (OSFED) or unspecified feeding / eating disorder (UFED) according to the *Diagnostic and statistical manual of mental disorders – fifth edition* (DSM-5) (6)
- No imminent risk of suicide. The therapist may choose to assess the risk of suicide using a structured mental health diagnostic interview, such as the *Mini-international neuropsychiatric interview* (7). If the participant presents a high risk of suicide, referral to a mental health treatment plan should be provided, and participation in the HAPIFED Program should be postponed until the participant no longer shows high risk of suicide.
- Accepts and is at least reasonably comfortable with attending therapy in a group format, which involves sharing personal problems with eating and weight, and listening to the difficulties of others, in a group setting.
- Is available to attend regularly (and not miss more than 3 sessions).
- Has a positive attitude towards daily recording of eating behavior, as this is an essential part of therapy.
- Has a positive attitude towards performing homework task that will be explained by the therapist.

If the potential participant meets all of the above eligibility criteria, then the therapist can proceed to the second aim of the session:

To build rapport, understand the participant's difficulties and help to increase motivation

- Assess the participant's current eating behaviors (e.g. eating habits, methods of shape and weight control, views on body image and weight), and assess any impairments resulting from the eating problem.
- Help the participant to reflect on what he/she would like to be different with regards to his/her eating behavior, body weight and shape.
- Assess general mental health condition (the therapist may choose to use a structured mental health diagnostic interview, such as the *Mini-international neuropsychiatric interview* (7), for this purpose).

- Assess the participant's history with previous eating disorder treatments and weight loss treatments, including what did or did not work well.
- Assess the participant's level of social support (e.g. from family or friends).
- Assess the participant's medical history, in order to assess whether current or past health conditions may impact upon their involvement in the program.
- Assess the participant's reasons for seeking treatment for his/her eating and weight difficulties, and what they hope to achieve through therapy.
- Explain to the participant the aims of the HAPIFED Program, which are:
    1. To promote strategies for healthy weight management (eating varied and nutritious foods according to physical hunger, combined with healthy physical activity).
    2. To reduce binge eating, self-induced vomiting and other eating disorder behaviors.
    3. To promote healthy self-evaluation (not dominated by concerns related to food, eating, weight or shape).

It is recommended that the therapist takes notes of relevant information obtained during the individual assessment, because this information may be useful for implementing individualized interventions during the program. The therapist should also assess if it may be necessary to refer the participant to other treatment (e.g. from a general practitioner or social worker) in addition to the HAPIFED Program.

Stage 1: Introduction and psychoeducation

Session 1: Introduction

(Stage 1: Introduction and psychoeducation)

Material to be distributed at this session

- *HAPIFED Diary* (the content of the *HAPIFED Diary* is described below in this session)
- Psychoeducation material: *The don't go hungry diet* by Amanda Sainsbury-Salis (2)

Introduction

In this session the therapist provides information about the HAPIFED Program and assesses and explores the participants' attitudes to the program. It is recommended that the therapist undertakes the following:

- Introduce himself / herself to the group of participants.
- Highlight the name of the program (HAPIFED) and explain what it stands for (A **H**ealthy **AP**proach to we**I**ght management and **F**ood in **E**ating **D**isorders).
- Remind participants of the aims of the HAPIFED Program, which are:
  1. To promote strategies for healthy weight management (eating varied and nutritious foods according to physical hunger, combined with healthy physical activity)
  2. To reduce binge eating, self-induced vomiting and other eating disorder behaviours
  3. To promote healthy self-evaluation (not dominated by concerns related to food, eating, weight or shape)
- Outline the number, dates, and times for the sessions.
- Explain the characteristics of the sessions (i.e. that it is a therapy based on talking within a group with a therapist, that all information divulged within the group is confidential, and that there will be homework tasks that participants will be expected to do between sessions).
- Explain the 3 stages of the HAPIFED Program.
- Encourage participants to ask questions throughout the program.
- Explain the disadvantages of not attending regularly or not finishing the program (e.g. missing important parts of the program).
- Invite participants to voice any concerns about the HAPIFED Program, and share their expectations about the program with the group.
- Ask participants about practicalities – how long it takes them to get to the sessions; anticipated difficulties such as holidays, and other potential barriers to engagement and program completion. Any identified barriers may be addressed in or out of session.

Psychoeducation about weekly weighing

In this activity, the therapist is to educate participants about the recommended frequency of weighing (once a week) and the rationale behind this (1). It is advised that the therapist sets up the group meeting room to be able to weigh participants once a week before or after the session, and to tell participants that they will be invited to be weighed once a week. It is important that the therapist emphasizes the following potential benefits of weekly weighing:

- It provides week-by-week feedback to participants about the impact of any changes in their eating or exercise habits on their weight.
- It provides an opportunity for the therapist to understand how participants feel about their weight or changes in their weight, and provide appropriate support.
- It is an opportunity for the participant to understand how a variety of factors (e.g. hydration levels, bowel and bladder state, menstrual cycle) affect their weight.
- It establishes an appropriate frequency of weight checking (once a week). Participants are asked not to weigh themselves at home while they are on the HAPIFED Program due to the possibility of preoccupation with weight and misinterpretation associated with inconsequential fluctuations that are more frequently observed when weighing more frequently than once a week.
- Almost all participants are concerned about the effect of the program on their weight. One aim of the HAPIFED Program is to give participants a sense of control over eating and – as much as is reasonable to expect – over body weight as well. Please note that participants should defer deciding upon a specific target weight range, if at all, until the end of program when their eating habits have stabilised. It is also recommended that the therapist explains that the HAPIFED Program promotes a healthy long-term change in eating behaviour and is not a 'quick fix' for excess weight.

HAPIFED formulation of loss of control over eating and weight gain

In this activity, the therapist is to explain the *HAPIFED formulation of loss of control over eating and weight gain* (Handout 1), discuss participants' thoughts on this formulation, and resolve any misunderstandings. It is recommended that the therapist explains that the *HAPIFED formulation of loss of control over eating and weight gain* is a general understanding of loss of control over eating and weight gain, and that it may need to be modified for each participant at a later date, as the eating behaviours and difficulties of each participant become more apparent.

Self-monitoring of eating behaviour

In this activity, the therapist is to explain that self-recording of eating behaviour is an essential part of the HAPIFED Program. A suggested way for the therapist to convey this to participants is as follows:

- Provide the HAPIFED Diary to participants. The HAPIFED Diary is a booklet (Handout 1) with the following material:

    1. Aims of the HAPIFED Program
    2. The *HAPIFED formulation of loss of control over eating and weight gain*
    3. *Instructions for self-monitoring* & *Hunger and satiety ratings*
    4. Examples of completed *HAPIFED monitoring forms*
    5. Enough *HAPIFED monitoring forms* for two weeks of treatment (14 copies)
    6. *Bi-weekly monitoring worksheet*

We recommend that a new HAPIFED Diary is provided to the participants for every two weeks of treatment.

- Explain *Instructions for self-monitoring* (Handout 1) and *Examples of completed monitoring forms* (Handout 1).
- Explain that there will be a bi-weekly monitoring of participants' eating behaviour records. This will occur in order to help participants to "keep on track" with healthy eating and physical activity behaviours, and to understand any difficulties they may experience during the program. More detailed information in regards to the bi-weekly monitoring will be provided in Session 3 (when the first bi-weekly monitoring will occur).
- Provide a copy of the *HAPIFED Summary sheet* (Handout 2) to participants and explain that the use of this material will provide an informative overview of participants' progress with the treatment.

Homework

- Start recording / self-monitoring eating behavior and physical activity, and aim to score all -2's or -3's in the Hunger column, and all +2s or +3s in the Satiety column.
    - Remind participants to bring their *HAPIFED Diary* (Handout 1) *and HAPIFED summary sheet* (Handout 2) to the next session.
- Practice not weighing at home.
- Read psychoeducation material (we suggest that participants read up to chapter 5 of Sainsbury-Salis, A. *The don't go hungry diet*. Australia: Bantam; 2007, because this is an introduction to the behavioural weight loss component of HAPIFED) (2). Note: *this material includes information on the physiology of weight loss, eating according to physical hunger and satiety signals, and issues around speed of weight loss with treatment.*

Handout 1: HAPIFED Diary

Aims of the HAPIFED (A **H**ealthy **AP**proach to we**I**ght management and **F**ood in **E**ating **D**isorders) **Program:**

1) To promote strategies for healthy weight management (eating varied and nutritious foods according to physical hunger, combined with healthy physical activity).

2) To reduce binge eating, self-induced vomiting and other eating disorder behaviours.

3) To promote healthy self-evaluation (not dominated by concerns related to food, eating, weight or shape).

30 Group Session Program Manual © da Luz, Swinbourne, Hay, Touyz, Palavras, Claudino, Sainsbury 2017

The HAPIFED formulation of loss of control over eating and weight gain

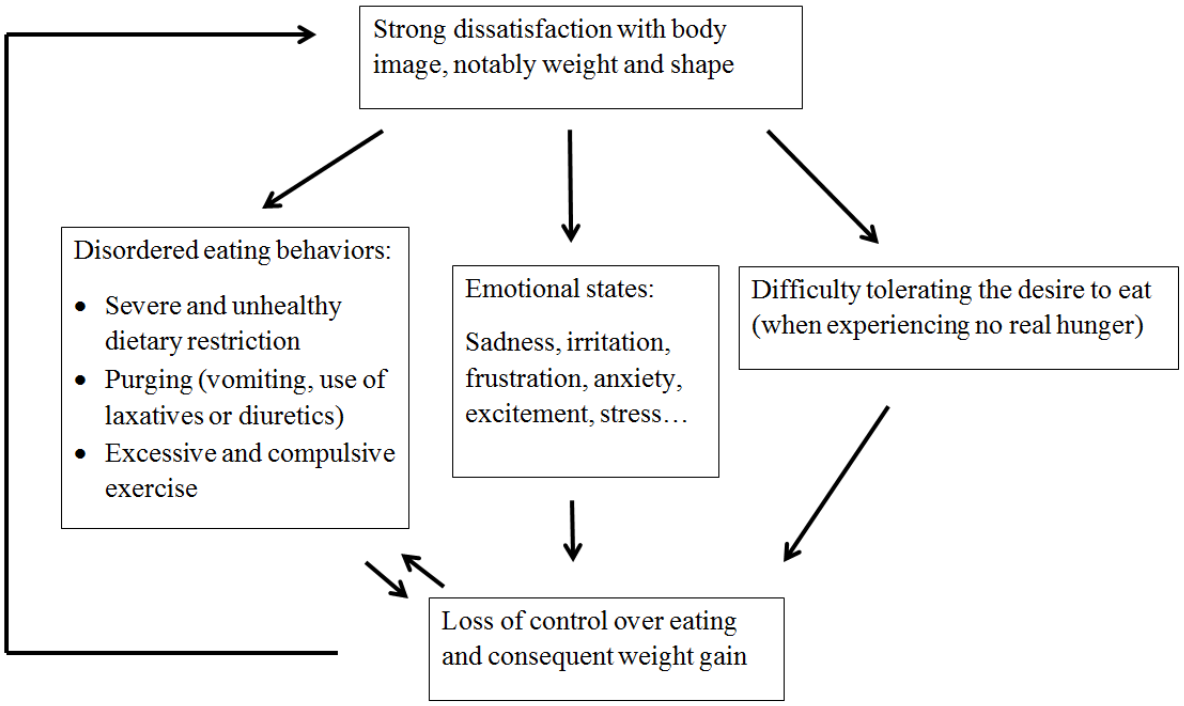

30 Group Session Program Manual © da Luz, Swinbourne, Hay, Touyz, Palavras, Claudino, Sainsbury 2017

Instructions for self-monitoring

(Adapted from *Cognitive behavior therapy and eating disorders* by Christopher Fairburn, (1), page 61, and *The Don't go hungry diet* by Amanda Sainsbury-Salis (2), pages 99-135.)

Self-monitoring is necessary for achieving the healthy eating habits promoted in the HAPIFED Program. It helps you and your therapist to understand your eating behavior in detail. Self-monitoring will enable you to become more aware of the following factors, every time you eat or drink (1):

- The quantity and quality of the foods and beverages that you are consuming.
- The time that you are spending eating or drinking, and the time since you last ate or drank something.
- The emotional, psychological, social and environmental circumstances that influence your consumption.

Your increased awareness of the above factors will facilitate appropriate changes in your eating habits.

How to use each of the following columns in the HAPIFED monitoring form (please see the example and blank forms on subsequent pages):

- Time: Record the time you started to eat and/or drink.
- Hunger: rate the level of hunger or satiety you felt *before eating or drinking*, from – 4 to +4 (using the hunger and satiety ratings scale provided).
- Food and drink consumed: record everything that you eat or drink, with the exception of foods or drinks that contain no or negligible kilojoules.
- Satiety: rate the level of hunger or satiety you felt *after eating*, from – 4 to +4 (also using the hunger and satiety ratings scale provided).
- Place: record where you were when you ate and/or drank.
- *: Place an asterisk adjacent to any episode(s) of eating or drinking that you feel was too large and/or if you felt that your eating was out of control.

- V/L/D (number): write “V” if you vomited, as well as the number of times you vomited, write “L” if you took laxatives, as well as the number consumed, or write “D” if you took water tablets (diuretics), as well as the number consumed.
- Context: record any events, and your thoughts or feelings, around the times you eat and/or drink.
- Physical activity (minutes or steps): record any physical activity undertaken throughout the day and the amount in minutes (or step counts if using a pedometer). This can include incidental activity (such as walking at the shops or working around the home), or formal exercise (attending a gym class, for example).

It is important that you:
Record what you consume, both food and kilojoule-containing drinks (but not kilojoules or calories). You do not need to record food or drinks that contain no or negligible kilojoules (e.g. a stick of sugarless chewing gum, plain water or mineral water, diet cordial, black tea or coffee that is unsweetened or artificially sweetened, diet cola).
- Aim to score all -2’s or -3’s in the Hunger column, and all +2s or +3s in the Satiety column.
- Record what you consume as close as possible to the time you ate or drank.
- Use a new form for every day.

Hunger and satiety ratings

Adapted from *the Don't go hungry diet* by Amanda Sainsbury-Salis (2), pages 102 and 116.

**- 4**	**- 3**	**- 2**	**- 1**	**0**	**+ 1**	**+ 2**	**+ 3**	**+ 4**
Ravenously Hungry	Very Hungry	Quite Hungry	A Little Bit Hungry	No Feeling	Unsatisfied	Just Satisfied	Elegantly Satisfied	Over Satisfied
I could eat anything right now.	I'd like to eat something substantial now.	I'd like to eat something now, perhaps a snack or light meal.	I'd be comfortable to wait a while before eating.	I'm not hungry at all.	I still feel a bit hungry and I'd gladly eat something else right now.	My body is relaxed and comfortable. If I ate any more I would still feel comfortable, but I don't need any more.	My body is relaxed and comfortable. If I ate any more I would begin to feel oversatisfied.	I know in my heart of hearts that I've eaten more than my body wants, and I feel uncomfortable.

HAPIFED monitoring form example 1

The following is an example of a day involving eating or drinking when not hungry or beyond the point of comfortable satiety. Note that you may eat more or less than the amounts shown in these examples. That is because hunger and satiety levels are different from one person to the next, and from one day to the next even in the same person.

Day of the week ……… Monday ……… Date …… 2nd May ……………

Time	Hunger	Food and drink consumed	Satiety	Place	*	V/L/D (number)	Context	Physical activity (minutes or steps)
7.30 am	-2	A couple of spoonfuls mango yogurt<br>4 poppyseed crackers with 1 laughing cow cheese<br>Cup tea	+2	Dining Room			Kept it small as thought I was having brunch at 10.30 am – this moved to lunch about 1 pm	
11 am	-1	Glass orange juice<br>2 poppy seed crackers<br>4 grapes<br>1 date	+2	At desk			Wanted energy before the gym	
11.40 am								60 minute workout at the gym
1 pm	-2	Vegetables and bocconcini on Turkish toast<br>1 spice cookie with cup tea	+3	At desk				
	1	Grazed throughout the afternoon on crackers, yogurt, grapes and dates	+ 4		*		Annoyed and disgusted at myself – why do I do this??	
7.30 pm	0	Slice of rye bread with butter<br>Caesar salad with grilled chicken<br>½ chocolate pancake	+2	Restaurant			Dinner out; trying to be good	
9.00 pm	0	2 cruskits with chevre & jam<br>3 spice cookies<br>Large handful poppy seed crackers<br>Tub yoghurt<br>2 pieces toast with peanut butter<br>1 bowl cereal	+4	Kitchen<br>Living Room	*	V (1)	Was in a bad mood after a bad day at work…craving chocolate but didn't have any so had this instead. Felt sick and disgusted with myself. The only good thing was that I stopped earlier than usual.	

*Consumption was excessive and/or with a sense of loss of control.

HAPIFED monitoring form example 2

The following is an example of eating and drinking according to hunger and satiety levels (i.e. all -2s or -3s in the Hunger column, and all +2s or +3s in the Satiety column). Note again that you may eat more or less than the amounts shown in the following example, because hunger and satiety levels are different from one person to the next, and even from one day to the next in the same person.

Day of the week …………… Friday …………………… Date ……….. 16th October ………………………

Time	Hunger	Food and drink consumed	Satiety	Place	*	V/L/D (number)	Context	Physical activity (minutes or steps)
8 am	-2	Small bowl red berry special K with raspberry/cranberry yoghurt<br>2 figs	+2	Dining Table				
12.30 pm	-3	Spinach, artichoke, carrot, sweet corn and olive salad with thousand island dressing	+2	Work kitchen				
4.30 pm								30 minute walk
5 pm	-2	Row (4 pieces) dark chocolate<br>Small bowl strawberries<br>Tea	+2	Dining table				
6.30 pm	-2	2 savory meatballs<br>Side roast eggplant<br>Side roast potatoes<br>Pint cider<br>Slice chocolate cake	+3	Pub			Went to the pub to celebrate my birthday. After dinner a friend bought a cake.	

*Consumption was excessive and/or with a sense of loss of control.

HAPIFED monitoring form

Day of the week ………………………………… Date …………………………………

Time	Hunger	Food and drink consumed	Satiety	Place	*	V/L/D (number)	Context	Physical activity (minutes or steps)

*Consumption was excessive and/or with a sense of loss of control.

Handout 2: HAPIFED summary sheet

What was your highest weight ever (in kg)? …………………………..…
On what date did you start this program? …………………………..…
What was your weight at the start of this program (in kg)? …………………………..…

**Data collected at the end of…**	Week 3	Week 5	Week 7	Week 9	Week 11	Week 13	Week 15	Week 17	Week 19	Week 21
**Date of data collection**										
**Your weight on the above date (kg)**										
**Change from the start of this program (kg)**										
**Change from your highest weight ever (kg)**										
Remember the 3 steps for weight loss. **Step 1.** Aim for *all* 2s and 3s in the Hunger and Satiety columns of your HAPIFED monitoring forms										
**Number of -1s or 0s in your Hunger column**										
**Number of +4s in your Satiety column**										
**Number of -4s in your Hunger column**										
**Number of +1s in your Satiety column**										
**Step 2.** Aim to enjoy 5 or more serves of vegetables and 2 or more serves of fruit *every* day										
**Average serves of vegetables per day**										
**Average serves of fruit per day**										
**Step 3.** Gradually increase the amount of physical activity you do to an average of 8,000 to 12,000 steps (or equivalent) per day										
**Average number of steps per day**										

30 Group Session Program Manual © da Luz, Swinbourne, Hay, Touyz, Palavras, Claudino, Sainsbury 2017

**Data collected at the end of…**	Week 23	Week 25	Week 27	Week 29	
**Date of data collection**					
**Your weight on the above date (kg)**					
**Change from the start of this program (kg)**					
**Change from your highest weight ever (kg)**					
Remember the 3 steps for weight loss. **Step 1.** Aim for *all* 2s and 3s in the Hunger and Satiety Columns of your HAPIFED monitoring forms.					
**Number of -1s or 0s in your Hunger column**					
**Number of +4s in your Satiety column**					
**Number of -4s in your Hunger column**					
**Number of +1s in your Satiety column**					
**Step 2.** Aim to enjoy 5 or more serves of vegetables and 2 or more serves of fruit *every* day.					
**Average serves of vegetables per day**					
**Average serves of fruit per day**					
**Step 3.** Gradually increase the amount of physical activity you do to an average of 8,000 to 12,000 steps (or equivalent) per day.					
**Average number of steps per day**					

Session 2: Psychoeducation about weight and behavioral weight loss
(Stage 1: Introduction and psychoeducation)

<u>Material to be distributed at this session</u>

- Psychoeducational material about binge eating disorder and bulimia nervosa (e.g. the first two chapters of Cooper P. *Overcoming bulimia nervosa and binge-eating: A self-help guide using cognitive behavioral techniques.* London: Robinson; 2009 (8). These chapters are entitled 1. What are binge-eating and bulimia nervosa? and 2. How binge-eating and bulimia nervosa affect people's lives)

<u>Check Homework</u>

- Review recording / self-monitoring of participants' eating behaviour and physical activity (the therapist reminds participants of the importance of having only 2s and 3s recorded in the Hunger and Satiety columns of their diary, of consuming fruit and vegetables every day in accordance with the prevailing national guidelines, and of the importance of gradually attaining and maintain physical activity levels of approximately 8,000 to 12,000 steps per day or its equivalent).

<u>Review of "Self-monitoring of eating behavior"</u>

In this activity, the therapist is to engage participants in self-monitoring of their eating behaviour. It is important that the therapist tries to obtain participants' perspectives and attitudes in regards to recording, and any difficulties that arose. Additionally, it is suggested that the therapist should address participants' experiences with the proposed behavioural weight loss strategy, the recording of their hunger/satiety before and after eating, and the wearing of a pedometer and recording physical activity or step counts. There are two important aspects that deserve the therapist's attention while conducting this review (adapted from Fairburn, C. *Cognitive behavior therapy and eating disorders.* New York: Guilford; 2008 (1)). These are to assess the quality of the recording, and to assess the information gained from the recording.

1. Assess the quality of the recording by determining…

- Whether recording was in "real" time.
- Whether asterisks and ratings of hunger and satiety have been used correctly.
- Whether any episodes of eating or drinking kilojoule-containing foods or beverages were not recorded.

Note: in this activity it is important that the therapist praises participants for any efforts in engaging in self-monitoring eating behaviour, even if they did not record all consumption since last session. It is also important that the therapist accepts that a participant may have not

commenced recording as yet, and to acknowledge and validate why this may be so (e.g. it is confronting and distressing to record binge and vomit episodes, it is a tedious activity, it is hard to fit into an already busy day). Participants who say that they forgot to bring their records should not be criticized, but instead encouraged with strategies to help them remember to bring them to the next session. However, it is also recommended that the therapist emphasize that recording – particularly real-time, accurate recording – is essential for the success of the HAPIFED Program.

2. Assess the information gained from the recording

In addition to assessing the quality of the recording, it is also recommended that the therapist assess the information gained from the recording about the participants' eating habits. It is suggested that the therapist asks the participants to briefly explain the information recorded in their diaries according to the following questions:

- Is the information recorded in your forms about typical or atypical days? Could you describe the differences in your eating behaviour from typical and atypical days?
- Did you record factors that may have motivated any behaviour(s) typical of eating disorders (e.g. binge eating, purging, excessive weighing etc.)?

Note: it is recommended that the therapist encourages participants to record anything that they feel could have some influence on their eating behaviour. It is also important that the therapist tries to clarify any information in the recordings that is not clear.

Psychoeducation about weight and behavioral weight loss

In this activity, the therapist is to review whether participants have read the psychoeducation material about behavioral weight loss (2), and to discuss participants' thoughts on the material. It is recommended that the therapist highlights to participants the importance of following three key behavioral weight loss strategies (2, 3):

1. Eating according to hunger and satiety cues.

2. Daily consumption of vegetables and fruits, in quantities recommended by the national nutrition guidelines of the country in which the HAPIFED Program is being implemented. In Australia, this is 5 or more servings of vegetables and 2 or more servings of fruits.

    - Aligning vegetable and fruit intake recommendations in HAPIFED with national recommendations helps to reduce conflicting messages about what to eat for health, and enables leveraging from existing national public health promotional activities around adequate vegetable and fruit intake, such as www.gofor2and5.com.au in Australia.

- It can be helpful to show material to demonstrate what this minimum daily intake of vegetables and fruit looks like. For example, one serve of vegetables in Australia is 75 g (about one cup of salad vegetables or half a cup of cooked vegetables, or a small potato). One serve of fruit in Australia is 150 g of fresh fruit (such as one medium pear or two apricots) or one cup of tinned, stewed or chopped fruit. It is also important that the therapist states that in order to lose weight, it is better if participants minimize juices, alcoholic beverages and dried fruits to four or fewer standard serves per week.

3. Aiming to get some form of physical activity every day, using a pedometer to monitor and record progress. Physical activity can be incidental (e.g. walking to the shops) or structured (e.g. going to a gym class or a brisk walk with the dog). Participants should be encouraged to monitor and then gradually increase the amount of physical activity they do, aiming eventually for the levels recommended for permanent weight loss (8,000 to 12,000 steps per day or its equivalent). For activities where participants did not use a pedometer (e.g. swimming), they may like to use the following guide from http://www.10000steps.org.au/help/pa-converting-steps/) to determine how many steps that activity is equivalent to:

    - 10 minutes of moderate intensity activity = ~1,000 steps. Moderate intensity activity causes a slight, but noticeable increase in breathing and heart rate. It is possible to maintain a conversation during moderate intensity activity.
    - 10 minutes of high intensity activity = ~2,000 steps. High intensity activity causes people to "huff and puff", and talking full sentences between breaths is difficult.

<u>Homework</u>

- Participants are asked to read psychoeducational material about binge eating disorder and bulimia nervosa as listed on page 20.
- Participants are requested to continue recording / self-monitoring their eating behavior and physical activity.
    - Remind participants to bring their *HAPIFED Diary* (Handout 1) *and HAPIFED summary sheet* (Handout 2) to the next session.

Session 3: Psychoeducation about eating disorders
(Stage 1: Introduction and psychoeducation)

Material to be distributed at this session

- Handout 3: *Bi-weekly monitoring worksheet*
- Handout 4: *Case vignettes*

Check homework

- Review recording / self-monitoring of participants' eating behaviour and physical activity (the therapist reminds participants of the importance of having only 2s and 3s recorded in the Hunger and Satiety columns of their diary, of consuming fruit and vegetables every day in accordance with the prevailing national guidelines, and of the importance of gradually attaining and maintain physical activity levels of approximately 8,000 to 12,000 steps per day or its equivalent).
- Review participants' understanding and thoughts on the psychoeducation material on bulimia nervosa and binge eating disorder that was recommended at the last session.

Psychoeducation about eating disorders

In this activity, the therapist is to discuss detailed information regarding binge eating disorder and bulimia nervosa with participants. It is advised that the therapist encourages participants to share their thoughts about this information, and to discuss whether they have developed a greater understanding of their own eating behavior from reading the provided information or listening to the discussion at this session. It is necessary that participants are informed about the following key points from the psychoeducational material (8):

- Definitions of binge eating disorder and bulimia nervosa.
- Common triggers of binge eating, purging and fasting.
- "Positive" and negative effects of binge eating, purging and fasting (including health consequences).
- Dysfunctional attitudes to weight, shape and eating.

Note: other psychoeducational material or resources besides the recommended text (8) can be used in this program, provided that it covers the key points outlined above.

Bi-weekly monitoring of participants' progress with behavioral weight loss therapy

In this activity, the therapist's aims are the following:

- Encourage participants to improve the quality of their recording of eating behavior.
- Assess participants' progress with the program.
- Identify any difficulties or barriers to change, and assist with strategies to resolve these.

The *Bi-weekly monitoring worksheet* (Handout 3) is introduced to participants and the monitoring is done in session. The therapist should remind participants to transfer their data from the *Bi-weekly monitoring worksheet* to their *HAPIFED summary sheet* (Handout 2), and any emerging patterns should be discussed in order to assist participants in achieving the goals of eating within hunger and satiety levels of ±2 and ±3, achieving adequate intake of fruit and vegetables, and achieving physical activity levels commensurate with long-term successful weight loss (8,000 to 12,000 steps per day or its equivalent).

Homework

- Participants continue recording / self-monitoring their eating behavior and physical activity.
    - Remind participants to bring their *HAPIFED Diary* (Handout 1) *and HAPIFED summary sheet* (Handout 2) to the next session.
- Participants are asked to read the *Case vignettes* (Handout 4), as a continuation of the psychoeducation on binge eating disorder and bulimia nervosa.

Handout 3: Bi-weekly monitoring worksheet

Summary of *The don't go hungry diet* (2) and *Don't go hungry for life* (3) by Amanda Sainsbury-Salis.

Losing excess weight and keeping it off by listening to your body is deceptively simple. 'All' you need to do is to eat mostly nutritious foods in accordance with your physical needs, and to do some form of regular physical activity.

Although this method of weight management is simple, and although you won't ever have to resist physical hunger in order to achieve it, that doesn't necessarily mean it's easy! Losing excess weight and keeping it off requires conscious attention.

To help yourself to stay focused and on track for success, use your HAPIFED Diary every day. After every two weeks, complete both sides of this bi-weekly monitoring worksheet.

After you have completed this worksheet, the therapist will show you how to use your results to understand and tweak your personal patterns so that you can lose weight as efficiently as possible.

By observing changes in your results from this worksheet over time, you will gain invaluable knowledge that will help you to achieve weight loss.

1. Let's review how you're going in using your HAPIFED Diary every day and aiming for all 2s and 3s in your Hunger and Satiety columns. This is the most important part of weight loss success.

The 'acid test' for knowing how well you're doing is to select the ***seven most recently completed pages (days)*** from your HAPIFED Diary from the past two weeks. Make a mark (e.g. an asterisk) on the corner of each of these seven pages so you know which ones to look at. Then go through those seven days and…

- Put a *circle* around any -1s or 0s in your Hunger column
- Put a *circle* around any +4s in your Satiety column
  (Circles represent times when you're eating more than you need)
- Put a *square* around any -4s in your Hunger column
- Put a *square* around any +1s in your Satiety column
  (Squares represent times when you're eating less than you need)

Write your results in the grey boxes in the table below. Also write these numbers into your *HAPIFED summary sheet* (Handout 2).

**Number of -1s or 0s in your Hunger Column**<br>(Nº circles in Hunger Column in 7 days)	…........	(aim for 2 or fewer per week)
**Number of +4s in your Satiety Column**<br>(Nº circles in Satiety Column in 7 days)	……..….	(aim for 2 or fewer per week)
**Number of -4s in your Hunger Column**<br>(Nº squares in Hunger Column in 7 days)	…........	(aim for 2 or fewer per week)
**Number of +1s in your Satiety Column**<br>(Nº squares in Satiety Column in 7 days)	…........	(aim for 2 or fewer per week)

After listening to the discussion about this during the session, what could you do differently to ensure that you are scoring almost *all* 2s and 3s in the Hunger / Satiety columns in your HAPIFED Diary?

………………………………………………………………………………

………………………………………………………………………………

………………………………………………………………………………

………………………………………………………………………………

………………………………………………………………………………

2. Now let's review how you're going in 'eating mostly nutritious foods'. The easiest way to achieve this is to focus on enjoying veggies and fruits every day.

The 'acid test' to see if you're eating enough veggies and fruits for sustainable weight loss is to pick the ***two most recently completed pages (days)*** from your HAPIFED Diary and review what you ate.

- Put a *tick* next to every serve of vegetables you ate
- Put a *star* next to every serve of fruit you ate

Remember, a serve of vegetables is 75 grams, or one cup of salad, or half a cup of cooked vegetables, or a small potato. A serve of fruit is 150 grams of fresh fruit, such as a medium-sized apple or pear, or two smaller pieces of fruit such as apricots or mandarins, or one cup of tinned, stewed or chopped fruit.

Write your results in the grey boxes in the table below. Also write these numbers into your *HAPIFED summary sheet* (Handout 2).

**Average serves of vegetables per day**<br>(Nº ticks in Food and drink consumed column in 2 days) ÷ 2	……..….	(aim for 5 or more per day)
**Average serves of fruit per day**<br>(Nº stars in Food and drink consumed column in 2 days) ÷ 2	……..….	(aim for 2 or more per day)

Since the start of this program, what are you doing well in the veggie and fruit department?

……………………………………………………………………………………….………..….

What – if anything – could you "tweak" (modify) in terms of your veggie or fruit intake to increase your intake to – or maintain it at – five plus two or more serves per day (which will help to ensure efficient weight loss)?

……………………………………………………………………………………………………

Points for discussion

- Think of maximum taste for veggies / fruit, not necessarily minimum fat / sugar
- Fun foods and alcohol: getting a sustainable balance that works
- Eat rather than drink your veggies and fruits whenever possible

3. Finally, let's review how you're going in doing some form of regular physical activity.

Look at the ***seven most recently completed pages (days)*** from your HAPIFED Diary.
Add up the total number of steps you did in those 7 days. For activities where you did not use a pedometer (e.g. swimming), use the following guide from http://www.10000steps.org.au/help/pa-converting-steps/) to determine how many steps that activity is equivalent to.

- 10 minutes of moderate intensity activity = ~1,000 steps
  Moderate intensity activity causes a slight, but noticeable increase in breathing and heart rate. It is possible to maintain a conversation during moderate intensity activity.
- 10 minutes of high intensity activity = ~2,000 steps
  High intensity activity causes people to "huff and puff", and talking full sentences between breaths is difficult.

Now, divide this total number of steps in those 7 days by 7. This is your average daily step count.
Write this number in the grey box below. Also write this number into your *HAPIFED summary sheet* (Handout 2).

**Average number of steps per day**	……..….	(aim to increase to 8-12K per day)

Since the start of this program, what are you doing well with your physical activity patterns?

…………………………………………………………………………………….………..…..

What – if anything – could you "tweak"(modify) in order to gradually increase to – or maintain your activity level at – 8,000 to 12,000 steps per day (which will help to ensure efficient and lasting weight loss)?

……………………………………………………………………………………………………

Points for discussion

- There is no 'right or wrong' way to be physically active for weight management. The most important thing is to find activities that you like enough to keep doing.

Handout 4: Case vignettes

Evan

Evan is 44 years old, married with one young child. He works as a project manager for a large building company. He has been overweight since he was a child and most of his family is also overweight. Although he was very sporty throughout school, after he left school and started working he stopped playing sport due to his work hours. His weight continued to increase, particularly after getting married and since the birth of his child.

Evan was not particularly concerned about his weight until his wife complained about his snoring and restlessness during the night and he went to see his doctor. His doctor told him he needed to lose weight as he thought he may have sleep apnea. His blood pressure was also dangerously high.

Evan started dieting strictly and going to the gym every day and after 2 weeks he had lost weight. He was pleased with his efforts and decided he would continue.

After a particularly stressful day at work he stopped off at the service station on his way home and bought a large amount of chocolate and chips. He ate this quickly on his way home and initially he felt a sense of relief and pleasure, however later he felt disgusted and angry with himself. He resolved to go back to strict dieting the next day.

Evan found himself repeating the same pattern of strict dieting followed by eating a large amount of "forbidden" food every time he experienced stress or disappointment. This happened more and more regularly and he started to feel his eating was out of control. He also noticed that his weight was increasing.

Evan felt ashamed of his eating behavior and he kept it a secret from everyone, even his wife. He also stopped exercising due to his weight gain and feelings of failure. He started to make excuses to avoid going to social events, something he had previously enjoyed.

Evan's wife started to notice his change in behavior and that he seemed irritable and stressed and encouraged him to tell her what was going on. After their conversation Evan's wife suggested that he might benefit from seeing a psychologist and Evan reluctantly agreed.

Emma

Emma is a 33-year old single woman who works in business administration. When she was a teenager she was a successful gymnast and performed at a competitive level. During this time she undertook rigorous training and was also highly restrictive in her diet in order to maintain her low weight. During that time she very occasionally found she was unable to resist overeating foods she enjoyed, and to compensate she would make herself vomit and fast afterwards.

After Emma started university she was unable to maintain her strict exercise and diet regimen and gradually gained weight. Emma felt extremely distressed and was very unhappy with her body and as a result tried even harder to restrict her eating. Emma was able to maintain strict dieting and daily exercise for a number of weeks, however as her university workload increased she found it impossible to maintain this routine. If her routine was disrupted in any way (such as being unable to exercise or unable to maintain her strict diet) she would find herself consuming large amounts of energy dense food (such as cakes, biscuits and chocolate) until she felt sick. After these episodes, Emma would make herself vomit. However as this became more regular and she found it increasingly difficult to make herself sick, she tried to fast after eating in an attempt to avoid weight gain. Emma's weight continued to increase and she became overweight.

Emma's extreme dissatisfaction with her weight led to a pattern of highly restrictive eating followed by uncontrolled overeating, vomiting or fasting. This continued for many years. Although she felt ashamed and embarrassed by her eating behavior, after noticing blood in her vomit she felt that she needed help to stop this pattern. She decided to see her doctor.

30 Group Session Program Manual © da Luz, Swinbourne, Hay, Touyz, Palavras, Claudino, Sainsbury 2017

Session 4: Nutritional education and problem solving
(Stage 1: Introduction and psychoeducation)

Material to be distributed at this session

- Nutritional education material
- Handout 5: *Proactive problem solving sheet*

Check homework

- Review recording / self-monitoring of participants' eating behaviour and physical activity (the therapist reminds participants of the importance of having only 2s and 3s recorded in the Hunger and Satiety columns of their diary, of consuming fruit and vegetables every day in accordance with the prevailing national guidelines, and of the importance of gradually attaining and maintain physical activity levels of approximately 8,000 to 12,000 steps per day or its equivalent).
- Therapist discusses participants' thoughts and reaction to reading the *Case vignettes* (Handout 4). Therapist discusses the material to answer any questions and clarify any potential misunderstandings.

Nutritional education

The aim of this activity is to provide important information to participants' in regards to:

- The fact that when we eat healthy foods in accordance with the body's hunger and satiety levels, increase our levels of physical activity and use a food and activity diary (such as the HAPIFED Diary), it leads to weight loss because it generally leads to the consumption of fewer kilojoules than the number of kilojoules than the body uses.
- Dietary recommendations for health, in accordance with the prevailing national dietary guidelines (e.g. Australian Dietary Guidelines (9)).
- Alcohol and weight loss.
- Energy density of foods.
- Portion distortion.
- The importance of protein and meal planning using the healthy plate model (¼ protein-rich foods, ¼ carbohydrate-rich foods, ½ low energy vegetables).

Note 1: it is recommended that the nutritional education not be prescriptive about what and how much to eat in order to lose weight, and not suggest eliminating any foods or beverages (e.g. alcohol or discretionary foods). Rather, the nutritional education should focus on the quantities and relative proportions of foods from different food groups that are required for overall general health. For example, it is important to consume a certain minimum amount of foods high in protein in order to maintain healthy muscle mass and bone density.

Note 2: it is recommended that the nutritional education be provided by a qualified nutrition professional (e.g. an Accredited Practicing Dietitian (Australia), registered dietitian or equivalent).

Proactive problem solving

In this activity, the therapist is to introduce and explain the step-by-step proactive problem solving technique to participants (Handout 5). The therapist can then ask participants to practice proactive problem solving as homework, starting with any difficulties that they may have identified in eating in accordance with the nutritional guidelines presented in this session. Furthermore, it is advised that the therapist encourages participants to use proactive problem solving as a strategy to address any current barriers to change.

Homework

- Participants continue recording / self-monitoring their eating behavior and physical activity.
    - Remind participants to bring their *HAPIFED Diary* (Handout 1) *and HAPIFED summary sheet* (Handout 2) to the next session.
- Participants are asked to engage in proactive problem solving to work on any difficulties they may have with eating for health (according to the information that was presented in this session) or resolving current barriers to change.

Handout 5: Proactive problem solving sheet

Step 1: Identify the problem as early as possible

Step 2: Specify the problem accurately

Steps 3 and 4: List as many possible solutions that you can, and think through the pros and cons of each possible solution

a)…………………………………………………………………………………………

(pros) ……………………………………………………………………………………

(cons) ……………………………………………………………………………………

b)………………………………………………………………………………………...

(pros) ……………………………………………………………………………………

(cons) ……………………………………………………………………………………

c) ………………………………………………………………………………………..

(pros) ……………………………………………………………………………………

(cons) ……………………………………………………………………………………

d) ………………………………………………………………………………………..

(pros) ……………………………………………………………………………………

(cons) ……………………………………………………………………………………

Step 5: Choose the best solution or combination of solutions

Step 6: Act on the solution

Step 7: Evaluate the process of problem solving

Example of proactive problem solving

Step 1: Identify the problem as early as possible
I don't have time to be active.

Step 2: Specify the problem accurately
I attempted to join a gym but I was the only member of the class who was overweight and wearing loose 'trackie" clothes. I was afraid of being observed exercising and being judged by others as unfit.

Steps 3 and 4: List as many possible solutions that you can, and think through the pros and cons of each possible solution

a) No change, continue to be inactive
(pros) Others won't judge me as unfit and I don't have to attend these awful gym classes.
(cons) I won't lose weight.

b) Joining a group planning an unsupported trek (carrying own bags and tents) to the base of Mt Everest
(pros) I will certainly lose weight!
(cons) This is too extreme. I'm not really prepared for that and I'll probably regain all the weight when I resume my routine.

c) Going for a moderately paced walk in usual day clothes in the evenings with a friend
(pros) That seems to be quite fun actually.
(cons) I'm not sure if I will find a friend to join me for the walks every time but I can try.

Step 5: Choose the best solution or combination of solutions
Option C is the best one.

Step 6: Act on the solution

Step 7: Evaluate the process of problem solving

Session 5: Physical activity education

(Stage 1: Introduction and psychoeducation)

<u>Material to be distributed at this session</u>

- Physical activity education material
- Handout 3: *Bi-weekly monitoring worksheet*
- Handout 6: *Healthy and unhealthy exercise*

<u>Check Homework</u>

- Review recording / self-monitoring of participants' eating behaviour and physical activity (the therapist reminds participants of the importance of having only 2s and 3s recorded in the Hunger and Satiety columns of their diary, of consuming fruit and vegetables every day in accordance with the prevailing national guidelines, and of the importance of gradually attaining and maintain physical activity levels of approximately 8,000 to 12,000 steps per day or its equivalent).
- Check if participants used proactive problem solving to work on any difficulties they may have with healthy eating, and to ask and discuss with participants what they may have noticed from this activity. It is advised that the therapist encourages participants to routinely use proactive problem solving to help promote healthy lifestyle habits.

<u>Bi-weekly monitoring of participants' progress with behavioral weight loss therapy</u>

In this activity, the therapist's aims are the following:

- Encourage participants to improve the quality of their recording of eating behavior.
- Assess participants' progress with the program.
- Identify any difficulties or barriers to change, and assist with strategies to resolve these.

The *Bi-weekly monitoring worksheet* (Handout 3) is again distributed to participants and the monitoring is done in session. The therapist should remind participants to transfer their data from the *Bi-weekly monitoring worksheet* to their *HAPIFED summary sheet* (Handout 2), and any emerging patterns should be discussed in order to assist participants in achieving the goals of eating within hunger and satiety levels of ±2 and ±3, achieving adequate intake of fruit and vegetables, and achieving physical activity levels commensurate with long-term successful weight loss (8,000 to 12,000 steps per day or its equivalent).

Physical activity education

The aim of this activity is to encourage participants to be physically active (including participation in some structured exercise), but in a healthy manner. It is recommended that the following issues regarding physical activity, exercise and health be addressed during this activity:

- Why exercise is important for health, including long-term weight management.
- Prevailing national physical activity guidelines.
- The fact that it takes more physical activity to maintain a reduced body weight than it takes to promote health and prevent weight gain in the first place. This means that the national physical activity guidelines should be seen as a minimum recommended level of physical activity.
- Strategies for incorporating physical activity into the day.
- The valuable role of incidental activity for weight management. Participants are encouraged to continue using pedometers to measure their level of physical activity, and to continue recording their activity level in their *HAPIFED Diary*.

Note: it is recommended that the physical activity education is provided by a licensed health care professional (e.g. a university-qualified exercise physiologist or equivalent).

Homework

- Participants continue recording / self-monitoring their eating behavior and physical activity.
    - Remind participants to bring their *HAPIFED Diary* (Handout 1) *and HAPIFED summary sheet* (Handout 2) to the next session.
- Participants are asked to read the examples and answer the questions in Handout 6 on *Healthy and unhealthy exercise*, and to bring this handout to the next session.

30 Group Session Program Manual © da Luz, Swinbourne, Hay, Touyz, Palavras, Claudino, Sainsbury 2017

Handout 6: Healthy and unhealthy exercise

Adapted from: Loughborough eating-disorder activity therapy “LEAP” cognitive-behavioural therapy for compulsive exercise in the eating disorders: therapist manual; *Lorin Taranis, Caroline Meyer, Stephen Touyz, Jon Arcelus and Michelle La Puma.* Unpublished material.

Introduction

What is healthy exercise? I think we would all agree that although some exercise is healthy, compulsive exercise is unhealthy, but how easy is it to correctly identify whether exercise is healthy or unhealthy? Detailed below you will find descriptions of six different exercisers. Read each description carefully and then decide whether the person described is a healthy exerciser or an unhealthy (compulsive) exerciser. Try to think about the factors that are maintaining their exercise behavior and then use the space below each description to record your reasons for labeling them healthy, or unhealthy.

Walking Wendy

Wendy is 34, works part-time at a shop and is a regular walker. Wendy always walks the same route at the same time every day, which only takes her about 45 minutes at a leisurely pace. Wendy's walk isn't motivated by weight or shape concerns as she never changes her walk if her diet changes and never tries to make up for any missed walks. In fact Wendy doesn't even break a sweat and never sets herself any exercise targets. However, Wendy does follow strict exercise rules. She cannot alter her route, or the time she goes for her walk, nor can she reduce the time or distance, or miss the walk for a day without feeling extremely anxious. Nor can Wendy tolerate being interrupted when she is walking as it makes her irritable and angry. Wendy doesn't feel any 'better' or 'happier' after her walk as she doesn't really enjoy it, she just feels less anxious.

Is Wendy a healthy exerciser or an unhealthy exerciser?

Healthy □ **Unhealthy □**

Reasons:

•	•
•	•
•	•
•	•

30 Group Session Program Manual © da Luz, Swinbourne, Hay, Touyz, Palavras, Claudino, Sainsbury 2017

Circuits Sam

Samantha is 27, a PhD student and is a fitness fanatic. She attends the same circuits class at a gym 5 times a week and goes for a run every day, at the same time each day, before she goes to university. She also does a yoga class at the weekend and will do extra gym classes if she has nothing else to do. Samantha always feels much happier after she has exercised and finds it really boosts her mood. Samantha believes that she needs to exercise every day in order to stay slim and that the fitter she is, the healthier she is. In order to make herself exercise as hard as she can, Samantha sets herself targets to reach at the gym, such as doing more repetitions or lifting more weight, and running targets such as running further in the same time or running the same distance faster. If she cannot reach one of her targets Samantha feels like she's let herself down and that her day has been ruined. Because Samantha believes that fitness and slimness are related she watches what she eats in order to not gain any weight and will often do more exercise to make up for eating a bad food or too much food. If Samantha cannot exercise for any reason she feels irritable and annoyed, and a bit low/depressed. She also worries that she will lose fitness and gain weight and so will make up for any exercise she has missed by doing more next time. Samantha often feels she isn't doing enough, or could be doing more exercise but it is difficult to fit in as it is already affecting her studies. If she has a lecture at the same time as her circuits class she will miss the lecture, and she often turns down invitations to parties as it will mean she misses an exercise session.

Is Samantha a healthy exerciser or an unhealthy exerciser?

Healthy □ **Unhealthy** □

Reasons:

Healthy	Unhealthy
•	•
•	•
•	•
•	•

Sporting Sue

Sue is 35, a full-time mum and is an active member of several sports teams. Sue never used to play any sport or do any other exercise, but she became concerned that she was putting on weight and decided to do some exercise to help her lose some weight. Sue initially joined a gym but found it too boring, so she joined her local women's soccer team. Sue found she enjoyed the social aspect of team sport a great deal and now plays soccer 3 times a week as well as playing tennis 2-3 times a week. Despite initially starting exercise to lose weight it is the social aspect and the enjoyment Sue gets from it that keeps her going. Sue also likes walking and often goes for long walks in the countryside or along the coast with her friends and family. Sue always feels a lot better after she has done some exercise and finds that it helps her to relax and unwind from a stressful day. Sue often misses one of her training sessions due to other commitments, or sometimes just because she can't be bothered, but so long as she makes it most of the time she feels that it is ok.

Is Sue a healthy exerciser or an unhealthy exerciser?

Healthy □ **Unhealthy □**

Reasons:

•	•
•	•
•	•
•	•

Random Rachel

Rachel is 20, is a music student and is a random exerciser. Rachel doesn't enjoy exercise. She always hated sport and is naturally not that active as a person. Rachel likes walking round the shops and doesn't mind walking to the train station or somewhere locally, but she doesn't enjoy gyms or exercise classes. Lots of Rachel's friends are regular exercisers being members of the gym and are very concerned about their weight and shape. Rachel didn't used to be bothered about her weight or shape as she is naturally quite slim albeit curvy, but since her friends are now all slimmer than her Rachel worries that she is fat. As a result Rachel often feels really guilty about the amount she has eaten and will do 1, 2, or even 3 hours of exercise to compensate. When Rachel feels the need to exercise she has to do it immediately even if she feels tired or has a prior commitment. If she cannot exercise Rachel feels guilty that she has let herself down. Rachel doesn't follow any sort of exercise routine and will often not exercise for several days, but when she does exercise she tends to do a lot, usually running, sit-ups and press-ups. Rachel's friends keep trying to get her to come to the gym with them, but she doesn't want to as she feels she would be the fattest person in the gym because all her friends are so thin. Rachel often joins her friends on various diets, but she finds them impossible to keep to and ends up exercising to make up for breaking the diet.

Is Rachel a healthy exerciser or an unhealthy exerciser?

Healthy □ **Unhealthy □**

Reasons:

Healthy	Unhealthy
•	•
•	•
•	•
•	•

30 Group Session Program Manual © da Luz, Swinbourne, Hay, Touyz, Palavras, Claudino, Sainsbury 2017

Marathon Michelle

Michelle is 42, works in an office and is training for a marathon. Michelle has always enjoyed running since she started cross country running at school when she was 12. Although she used to compete in local and national competitions Michelle does not compete any more. However, Michelle is very competitive by nature and highly perfectionistic, so she always strives to do the best she possibly can. She is following a strict training regime, which she got from a running magazine, that is specific for running a marathon and slowly builds up her weekly mileage. The training regime also incorporates weekly performance goals that should be aimed for. Although the training regime is strict, each week is different and always incorporates 2 or 3 rest days. Michelle follows the prescribed routine exactly and never tries to do any more. On average Michelle is doing about 7-8 hours of exercise a week. Michelle is also following a fairly strict diet. She weighs herself several times a week to make sure that she is maintaining her weight and she increases her calorific intake each week to make up for the extra exercise. Michelle is aware that her running keeps her slim and in good shape, and she also likes the way running makes her feel, but she knows that too much exercise can damage her health so several times a year she will stop all exercise for a week or two to give her body a rest. Although she misses running when she stops, Michelle doesn't worry or feel guilty and isn't concerned that she'll gain weight or lose shape.

Is Michelle a healthy exerciser or an unhealthy exerciser?

**Healthy □**	**Unhealthy □**
**Reasons:**	
•	•
•	•
•	•
•	•

30 Group Session Program Manual © da Luz, Swinbourne, Hay, Touyz, Palavras, Claudino, Sainsbury 2017

Active Annie

Annie is 29, works as a groom at a stable yard and is a regular gym user. Despite having a physical job Annie goes to the gym 5 times a week for an hour after work, in order to stay slim and in shape. Annie finds the gym a real chore as she doesn't really enjoy it, but feels she has to do it if she wants to stay slim. She makes herself go even though she is often exhausted after a hard day at work. If Annie is unable to go to the gym for whatever reason she always feels really guilty about missing it, like she's let herself down. She also worries that she will become unattractive if she doesn't keep going to the gym as she believes that she needs to exercise in order to be attractive. Annie is always looking at the other people in the gym, comparing herself to them and thinking that they are slimmer and more attractive than her. She also notices that they seem to have more energy than her and she thinks this is because they are fitter than her. Annie thinks that the slimmer you are the more attractive you are and that if she were slimmer she would feel a lot better.

Is Annie a healthy exerciser or an unhealthy exerciser?

Healthy □ **Unhealthy □**

Reasons:

•	•
•	•
•	•
•	•

Please bring this handout with you to the next session, as it will be needed.

Session 6: Healthy versus excessive exercise
(Stage 1: Introduction and psychoeducation)

There is no new material to be distributed at this session

Check Homework

- Review recording / self-monitoring of participants' eating behaviour and physical activity (the therapist reminds participants of the importance of having only 2s and 3s recorded in the Hunger and Satiety columns of their diary, of consuming fruit and vegetables every day in accordance with the prevailing national guidelines, and of the importance of gradually attaining and maintain physical activity levels of approximately 8,000 to 12,000 steps per day or its equivalent).
- Discuss with participants their responses to Handout 6 on *Healthy and unhealthy exercise*.

Healthy versus unhealthy exercise

In this activity, the therapist is to discuss with participants the concept of exercising in a healthy manner, and to discuss ideas on how they can incorporate this into their lives. The main points for discussion are:

- Just as with a sedentary life style, exercise in a compulsive manner is not healthy.
- The definitions of excessive or driven exercise, and compensatory or non-compensatory exercise (1).
- Negative health effects of excessive exercise (4): risk of pain and injuries, the time consuming nature of excessive exercise and the subsequent restriction of other activities, fatigue, chronic dissatisfaction with the amount of exercise performed, distress when unable to exercise in the same ritualistic manner, feeling compelled to exercise despite contraindications such as illness or injury), and the fact that excessive exercise can trigger disordered eating.
- The best reasons to exercise are to maintain good health and to have fun; not to develop the "perfect body".
- Psychological and social benefits of engaging in healthy exercise.

Note: the therapist needs to be careful not to discourage participants from engaging in healthy exercise, but should only discourage driven, compulsive and excessive exercise that is "unhealthy".

Homework

- Participants continue recording / self-monitoring their eating behavior and physical activity.
    - Remind participants to bring their *HAPIFED Diary* (Handout 1) *and HAPIFED summary sheet* (Handout 2) to the next session.
- In addition to the above, this week the therapist emphasizes that any “unhealthy” exercise episodes should be recorded (if they occur). This is important because it can potentially provide additional information to the participant and therapist in regards to triggers to any episodes of excessive exercise that a participant may experience.
- The therapist asks participants to try some form of healthy exercise that can be undertaken prior to the next session (e.g. attending some type of group exercise activity that they enjoy, planning a walk with a friend).

Session 7: Nutritional education (continued)
(Stage 1: Introduction and psychoeducation)

Material to be distributed at this session

- Handout 3: *Bi-weekly monitoring worksheet*

Check homework

- Review recording / self-monitoring of participants' eating behaviour and physical activity (the therapist reminds participants of the importance of having only 2s and 3s recorded in the Hunger and Satiety columns of their diary, of consuming fruit and vegetables every day in accordance with the prevailing national guidelines, and of the importance of gradually attaining and maintain physical activity levels of approximately 8,000 to 12,000 steps per day or its equivalent).
- Check if participant's engaged in any form of "unhealthy exercise".
- Check if the participants tried some form of healthy exercise (e.g. attending some type of group exercise activity that they enjoy, or going on a walk with a friend).

Bi-weekly monitoring of participants' progress with behavioral weight loss therapy

In this activity, the therapist's aims are the following:

- Encourage participants to improve the quality of their recording of eating behavior.
- Assess participants' progress with the program.
- Identify any difficulties or barriers to change, and assist with strategies to resolve these.

The *Bi-weekly monitoring worksheet* (Handout 3) is again distributed to participants and the monitoring is done in session. The therapist should remind participants to transfer their data from the *Bi-weekly monitoring worksheet* to their *HAPIFED summary sheet* (Handout 2), and any emerging patterns should be discussed in order to assist participants in achieving the goals of eating within hunger and satiety levels of ±2 and ±3, achieving adequate intake of fruit and vegetables, and achieving physical activity levels commensurate with long-term successful weight loss (8,000 to 12,000 steps per day or its equivalent).

Nutritional education with dietitian (continued)

Suggested topics:

- Review of key points from Session 4.
- Review of any changes in eating behavior that participants may have made in response to the nutritional information presented in the previous session of nutritional education.
- Address participants' questions.

Homework

- Participants continue recording / self-monitoring their eating behavior and physical activity.
    - Remind participants to bring their *HAPIFED Diary* (Handout 1) *and HAPIFED summary sheet* (Handout 2) to the next session.

30 Group Session Program Manual © da Luz, Swinbourne, Hay, Touyz, Palavras, Claudino, Sainsbury 2017

Stage 2: Core interventions

Session 8: Changes in eating related to events and moods
(Stage 2: Core interventions)

Material to be distributed at this session

- Handout 5: *Proactive problem solving sheet*
- Handout 7: *Reviewing a situation that triggered disordered eating*

Check Homework

- Review recording / self-monitoring of participants' eating behaviour and physical activity (the therapist reminds participants of the importance of having only 2s and 3s recorded in the Hunger and Satiety columns of their diary, of consuming fruit and vegetables every day in accordance with the prevailing national guidelines, and of the importance of gradually attaining and maintain physical activity levels of approximately 8,000 to 12,000 steps per day or its equivalent).

Changes in eating related to events and mood

In this activity, the therapist is to help participants to find effective ways to cope with changes in eating related to adverse events and emotional fluctuations. It is recommended that the therapist explains that it is very common for adverse events and changes in mood to trigger disordered eating (1). Next, the therapist can ask participants to engage in the following activities:

- Remember the last time you experienced an adverse event or emotional fluctuation that triggered disordered eating.
- Using the *Proactive problem solving sheet* (Handout 5), try to find a potential solution for the situation that would have reduced your chances of engaging in disordered eating.

In some cases, it is not possible to solve problematic events that trigger disordered eating, or some participants may be sensitive to unpleasant emotional states, leading to low tolerance for such states and engagement in disordered eating (1). In these cases, it is recommended that the therapist discuss with participants their experiences with situations when unpleasant emotional states triggered disordered eating, and the proactive problem solving exercise should focus on improving participants' emotional state.

Next, it is recommended that the therapist invites participants to read and respond to Handout 7 (*Reviewing a situation that triggered disordered eating*). After participants write answers to the questions in Handout 7, it is recommended that the therapist suggest to participants that they accept that they can experience unpleasant emotional states, because everyone experiences them, but to remind themselves that they are usually temporary states and that all emotions peak and subside over time, and that unpleasant emotions can be enhanced or reduced depending on what we do (for example, we can implement strategies to reduce distress, such as, writing about the situation, our thoughts and emotions; engaging in pleasant or self-soothing activities, or engaging in activities that generate a sense of achievement).

Finally, it is suggested that the therapist invite participants to think about and implement activities that reduce the likelihood of engaging in disordered eating behaviors when they experience negative moods, such as avoiding food triggers.

<u>Homework:</u>

- Participants continue recording / self-monitoring their eating behavior and physical activity.
    - Remind participants to bring their *HAPIFED Diary* (Handout 1) *and HAPIFED summary sheet* (Handout 2) to the next session.
- Participants are asked to pay close attention this week to any eating that is not driven by hunger (that is – any episodes of eating or drinking kilojoule-containing foods or beverages when not physically hungry), and to ask themselves what else could be influencing their eating (e.g. feelings, including physical sensations, or distressing thoughts) and write it down in detail in the Context column of their *HAPIFED Diary*, including on the back page if more space is required.
- Participants are asked to review Handout 7 (*Reviewing situation that triggered disordered eating*) at home, and to add or modify information they wrote on it if needed, and to bring this handout to the next session.

Handout 7: Reviewing a situation that triggered disordered eating

1. What was the situation that triggered disordered eating?

……

……

……

……

2. What were your feelings at that moment?

……

……

……

……

3. What triggered those feelings?

……

……

……

……

4. What were the thoughts going through your mind at that moment?

……

……

……

……

5. The next questions are designed to help you develop more functional thoughts about the situation (this is better done in writing). What is the evidence that the thoughts you wrote in 4, above, were true? What is the evidence that these thoughts were NOT true? Is there another way to think about that situation? What would a friend say about that situation?

……

……

……

……

30 Group Session Program Manual © da Luz, Swinbourne, Hay, Touyz, Palavras, Claudino, Sainsbury 2017

Session 9: Changes in eating related to events and moods (continued)
(Stage 2: Core interventions)

Material to be distributed at this session

- Handout 3: *Bi-weekly monitoring worksheet*
- Handout 8: *Action plan*

Check Homework

- Review recording / self-monitoring of participants' eating behaviour and physical activity (the therapist reminds participants of the importance of having only 2s and 3s recorded in the Hunger and Satiety columns of their diary, of consuming fruit and vegetables every day in accordance with the prevailing national guidelines, and of the importance of gradually attaining and maintain physical activity levels of approximately 8,000 to 12,000 steps per day or its equivalent).
- Review participants' experiences with paying close attention to any eating that was not driven by physical hunger, asking themselves what else could be influencing their eating (e.g. feelings, inducing physical sensations, distressing thoughts), and writing it down in detail in their *HAPIFED Diary*.
- Review if participants added or modified information written on Handout 7 (*Reviewing a situation that triggered disordered eating*), and discuss if appropriate.

Bi-weekly monitoring of participants' progress with behavioral weight loss therapy

In this activity, the therapist's aims are the following:

- Encourage participants to improve the quality of their eating behavior recording.
- Assess participants' progress with the program.
- Identify any difficulties or barriers to change and assist with strategies to resolve these.

The *bi-weekly monitoring worksheet* (Handout 3) is again provided to participants and the monitoring is done in session. The therapist should remind participants to transfer their data from the *Bi-weekly monitoring worksheet* to their *HAPIFED summary sheet* (Handout 2), and any emerging patterns should be discussed in order to assist participants in achieving the goals of eating within hunger and satiety levels of ±2 and ±3, achieving adequate intake of fruit and vegetables, and achieving physical activity levels commensurate with long-term successful weight loss (8,000 to 12,000 steps per day or its equivalent).

Changes in eating related to events and mood (continued)

In this activity, the therapist is to ask participants to deeply think about activities that have reduced the likelihood of their engagement in disordered eating behaviors in the past, and activities of this type that they think may be beneficial in the near future. Next, it is recommended that the therapist ask participants to write an *Action plan* using Handout 8 for the next time they feel tempted to engage in disordered eating

30 Group Session Program Manual © da Luz, Swinbourne, Hay, Touyz, Palavras, Claudino, Sainsbury 2017

behaviours. After participants write their actions plans, it is recommended that the therapist ask them to share their *Action plan* with the group, because their ideas may be useful to other participants.
If necessary, the therapist can review important points from the previous session (Session 8), such as:

- The importance of developing functional thoughts about situations that trigger disordered eating, as initiated in Handout 7 on *Reviewing a situation that triggered disordered eating* (this is better done in writing). The therapist can provide examples of useful questions to develop accurate and functional thoughts, such as: What is the evidence that this thought was true? What is the evidence that this thought was NOT true? Is there another way to think about the situation that triggered disordered eating? What would a friend say about that situation?
- The importance of participants accepting that they can experience unpleasant emotional states, because everyone experiences them, and of reminding themselves that they are usually temporary states and that all emotions peak and subside over time, and that unpleasant emotions can be enhanced or reduced depending on what we do (for example, we can implement strategies to reduce distress, such as writing about the situation, our thoughts and emotions, engaging in pleasant or self-soothing activities, or engaging in activities that generate a sense of achievement).

<u>Homework:</u>

- Participants continue recording / self-monitoring their eating behavior and physical activity.
    - Remind participants to bring their *HAPIFED Diary* (Handout 1) *and HAPIFED summary sheet* (Handout 2) to the next session.
- Participants are asked to review their *Action plan* (Handout 8) and to modify or change their plan if they see that it can be improved.

Handout 8: Action plan

The next time I feel tempted to engage in disordered eating behavior, namely (e.g. overeating, inducing vomiting, compulsive exercise, fasting…) …………………………………………..., instead of engaging in disordered eating behavior I can do one or a combination of the following activities:

1)………………………………………………………………………………………………
………………………………………………………………………………………………
………………………………………………………………………………………………
………………………………………………………………………………………………

2)………………………………………………………………………………………………
………………………………………………………………………………………………
………………………………………………………………………………………………
………………………………………………………………………………………………

3)………………………………………………………………………………………………
………………………………………………………………………………………………
………………………………………………………………………………………………
………………………………………………………………………………………………

4)………………………………………………………………………………………………
………………………………………………………………………………………………
………………………………………………………………………………………………
………………………………………………………………………………………………

5)………………………………………………………………………………………………
………………………………………………………………………………………………
………………………………………………………………………………………………
………………………………………………………………………………………………

Session 10: Mindfulness / Social environment

(Stage 2: Core interventions)

Material to be distributed at this session

- Handout 9: *Interpersonal conflicts* (participants should be asked to not look at the reverse side of the handout until after they have considered and discussed the activity on the front page)
- Handout 5: *Proactive problem solving sheet*

Check Homework

- Review recording / self-monitoring of participants' eating behaviour and physical activity (the therapist reminds participants of the importance of having only 2s and 3s recorded in the Hunger and Satiety columns of their diary, of consuming fruit and vegetables every day in accordance with the prevailing national guidelines, and of the importance of gradually attaining and maintain physical activity levels of approximately 8,000 to 12,000 steps per day or its equivalent).
- Review participants' experience with paying close attention to any eating that was not driven by hunger, asking themselves what else could be influencing their eating (e.g. feelings, including physical sensations, distressing thoughts) and writing it down in detail.
- Check if participants reviewed their *Action plan* (Handout 8) at home and made any improvements to it. It is recommended that the therapist provides positive feedback to participants that made improvements on their *Action plan* for homework.

Mindfulness exercise

In this activity, the therapist is to encourage participants to eat in a more mindful manner in order to improve awareness and reduce mindless eating. The exercise of eating a sultana in a mindful manner (10) is led by the therapist with participants during the session. In this exercise, participants are guided by the therapist in a step-by-step process to hold, observe, touch, smell, place in their mouth, taste, chew and swallow a sultana in a very slow, attentive and mindful manner (10). It is recommended that this mindfulness eating exercise last for approximately 15-20 minutes. Following this activity, the therapist debriefs the participants, asking them about their observations and insights. Participants are encouraged to practice this exercise at home by themselves with a variety of other foods.

Managing the social environment

In this activity, the therapist is to discuss with participants the involvement of relatives or friends in order to create an environment that promotes improvements in their eating behavior. The involvement of relatives or friends is indicated in two instances: 1) if the participant feels that they can help them to make positive changes or 2), if the participant feels that they interfere negatively with their recovery (1).

When participants identify a clear indication for involvement of relatives or friends, it is recommended that the therapist discuss with participants how to ask relatives or friends for support, including how to raise issues that interfere with therapy with family or friends. It is recommended that the therapist help participants improve their skills with the following two situations: 1) effectively request that others change their unhelpful behavior and 2) refuse inappropriate requests from others. Because reasonable, direct and empathic requests are more likely to result in appropriate behavior change from others (11), it is advised that the therapist uses the examples on the first page of Handout 9 (*Interpersonal conflicts*) to help participants develop their communication skills. After reading each example aloud to the group, the therapist can ask participants to role-play in pairs a reasonable, direct and empathic response to the comments in the example. After that, the therapist reflects and provides feedback to participants on their responses, and provides examples of appropriate responses using the reverse side of Handout 9.

Group Exercise

Ask participants to provide real-life examples of unhelpful behavior or inappropriate requests from others that has impacted on their eating behavior. It is recommended that the therapist encourages the group to brainstorm possible responses that are reasonable, direct and empathic, and then role-play these responses.

Note: if a participant feels that it is very unlikely that relatives or friends will change their behavior in a positive manner, it is advised that the therapist discusses with participants what they can do to create a better environment for themselves or be less affected by any harmful attitudes of relatives or friends. For example, refer participants to the structured problem solving exercise covered in session 4 (Handout 5).

Homework

- Participants continue recording / self-monitoring their eating behavior and physical activity.
  - Remind participants to bring their *HAPIFED Diary* (Handout 1) *and HAPIFED summary sheet* (Handout 2) to the next session.
- Participants are invited to practice mindful eating.
- Participants are asked to practice reasonable, direct and empathic requests to other people that may be interfering positively or negatively with their eating behavior.
- Participants are invited to use proactive problem solving in order develop a social environment with relatives and friends that is helpful for the improvement of their eating behavior, in accordance with what was discussed in this session.

Handout 9: Interpersonal conflicts

Scenario 1

Your partner is very critical when he notes that you have chocolate cookies in the kitchen cupboard. You have been seeing a dietician who has been encouraging you to eat a wide range of foods (including cookies occasionally). You bought the cookies to eat occasionally, and one at a time when you are really craving them. Your partner says: "I don't understand why you always do the wrong thing, you spend money and time seeing the dietitian and you don't even follow her advice! This is such a simple choice; you shouldn't eat chocolate cookies if you want to lose weight!"

Scenario 2

You are at the birthday party of your friend, which is a dinner at her house. There are different dishes on offer. Your friend insists that you should have the four-cheese lasagna and chips because they are delicious, however you prefer the baked fish with salad, which also look good and are healthier.

Scenario 3

You are due to finish work in 15 minutes and have planned to go for a walk and after that cook dinner. Unexpectedly, your boss asks you to stay longer in order to help with a difficult project.

Examples of appropriate responses to situations of interpersonal conflicts

Example of appropriate response to scenario 1

I know you want to help me with losing weight, but when you say that I always do the wrong thing I get angry and upset because it seems like you don't see the positive changes that I'm doing such as buying more fruits and vegetables. If you avoid saying critical comments like that and understand that I am trying my best, then I will feel less stressed and I will feel more confident to change my behavior and eat healthier.

Example of appropriate response to scenario 2

I know that the four-cheese lasagna and chips look really delicious and you would be happy if I tried them but I really prefer the fish and salad.

Example of appropriate response to scenario 3

I know you would like me to work back but I already have plans this evening. If you can meet me tomorrow morning then I will be able to help with the project and start earlier if necessary.

Session 11: Dissatisfaction with body weight and shape
(Stage 2: Core interventions)

Material to be distributed at this session

- Handout 1: *HAPIFED formulation of loss of control over eating and weight gain*
- Handout 3: *Bi-weekly monitoring worksheet*
- Handout 10: *Aim: Focus on positive aspects of your body*

Check Homework

- Review recording / self-monitoring of participants' eating behaviour and physical activity (the therapist reminds participants of the importance of having only 2s and 3s recorded in the Hunger and Satiety columns of their diary, of consuming fruit and vegetables every day in accordance with the prevailing national guidelines, and of the importance of gradually attaining and maintain physical activity levels of approximately 8,000 to 12,000 steps per day or its equivalent).
- Discuss any mindful eating experiences that the participants may have experienced in the past week.
- Review whether participants engaged in making any reasonable, direct and empathic requests of other people that may be interfering positively or negatively with their eating behavior.
- Check participants' use of proactive problem solving to develop a social environment with relatives and friends that is helpful for the improvement of their eating behavior, in accordance with what was discussed in the last session.

Bi-weekly monitoring of participants' progress with behavioral weight loss therapy

In this activity, the therapist's aims are the following:

- Encourage participants to improve the quality of their recording of eating behavior.
- Assess participants' progress with the program.
- Identify any difficulties or barriers to change, and assist with strategies to resolve these.

The *Bi-weekly monitoring worksheet* (Handout 3) is again distributed to participants and the monitoring is done in session. The therapist should remind participants to transfer their data from the *Bi-weekly monitoring worksheet* to their *HAPIFED summary sheet* (Handout 2), and any emerging patterns should be discussed in order to assist participants in achieving the goals of eating within hunger and satiety levels of ±2 and ±3, achieving adequate intake of fruit and vegetables, and achieving physical activity levels commensurate with long-term successful weight loss (8,000 to 12,000 steps per day or its equivalent).

30 Group Session Program Manual © da Luz, Swinbourne, Hay, Touyz, Palavras, Claudino, Sainsbury 2017

Dissatisfaction with body weight and shape

In this activity, the therapist is to review and discuss the *HAPIFED formulation of loss of control over eating and weight gain* (Handout 1) with the participants, emphasizing the key role that having a strong dissatisfaction with body weight and shape has in maintaining binge eating and consequent weight gain. It is advised that the therapist explains that some level of dissatisfaction with body image, namely weight and shape, is common to most people, however when this dissatisfaction is overwhelming and is associated with one or more of the following three situations, it can lead to binge eating and consequent weight gain:

- **Extreme and risky attempts to lose weight.** A strong dissatisfaction with body weight and shape can lead people to engage in desperate attempts to lose weight that are risky for their health. This can include but is not limited to severe dietary restriction without supervision by a healthcare professional, self-induced vomiting, misuse of laxatives and diuretics, and excessive exercise. Engaging in such behaviors can trigger subsequent binge eating for two reasons: the body is undernourished and this therefore leads to very strong hunger, and/or because the extreme and risky attempts to lose weight may seem like a "solution" to eliminate the excess kilojoules ingested during a binge, thereby "enabling" a person to binge eat as much as they want.

- **Emotional states such as sadness, irritation, frustration, anxiety, excitement and stress.** A strong dissatisfaction with body weight and shape, associated with certain emotional states, can lead people to look for pleasure in "comfort foods", or to eat mindlessly.

- **Difficulty tolerating the desire to eat (when experiencing no real physical hunger).** When people regularly eat because they cannot resist the taste or pleasure of eating certain foods that they like (and not because they need to satisfy their physical needs for nutrition), they will inevitably gain weight. Difficulty tolerating the desire to eat when experiencing no real physical hunger *per se* may be a sign of loss of control over eating.

The discussion above introduces the next activity (Liking your body), which aims to encourage development of a healthy body image.

Liking your body

The aim of this exercise is to help participants to develop a more positive attitude towards their body. This is important because individuals with eating disorders tend to focus their attention on the aspects of their bodies that they do not like. It is recommended that the therapist uses the first activity in Handout 10 in session with participants, and encourage a discussion on the importance of liking their own bodies.

Homework

- Participants continue recording / self-monitoring their eating behavior and physical activity.
    - Remind participants to bring their *HAPIFED Diary* (Handout 1) *and HAPIFED summary sheet* (Handout 2) to the next session.
- Therapist asks participants to practice the second exercise of Handout 10, entitled *Aim: Focus on positive aspects of your body*.

Handout 10: Aim: Focus on positive aspects of your body

(Adapted from Noordenbos, G. *Recovery from eating disorders: a guide for clinicians and their clients.* Oxford: Wiley-Blackwell; 2013, with permission from Professor Greta Noordenbos (12))

Most people with eating disorders focus only on the negative aspects of their bodies and ignore the positive sides. To develop a more positive body attitude, it is important to concentrate on positive aspects. In the following list we ask you to circle all those aspects of your body which are OK:

1. Hair
2. Forehead
3. Eyebrows
4. Eyes
5. Eyelashes
6. Nose
7. Ears
8. Chin
9. Teeth
10. Skin of your face
11. Neck
12. Shoulder
13. Upper arms
14. Lower arms
15. Hands
16. Fingers
17. Nails
18. Torso
19. Wrist
20. Waist
21. Upper back
22. Lower back
23. Bum
24. Thighs
25. Knees
26. Calves
27. Ankles
28. Feet
29. Toes
30. Toenails

How many body parts did you circle?

Now make a short list of those parts or your body that you like.

Focus on the positive parts every day

In this assignment you learn to evaluate your body in a more positive way and to pay your body compliments. The positive evaluation should be directed not only at your body appearance but can also focus on the function of your body. For example, "I like my hair and eyes. Today I went for a very nice walk with our dog and that was really fun".

Developing a positive attitude does not mean that you have to be completely satisfied with all aspects of your body. The aim is to develop a general feeling of accepting yourself and your body, even those aspects which might not be as perfect as you would want. You should write at least three positive things about your body in your notebook every day.

	Monday	Tuesday	Wednesday	Thursday	Friday	Saturday	Sunday
1							
2							
3							

Session 12: Dissatisfaction with body weight and shape (continued)
(Stage 2: Core interventions)

Material to be distributed at this session

- Handout 11: Identifying my good points

Check Homework

- Review recording / self-monitoring of participants' eating behaviour and physical activity (the therapist reminds participants of the importance of having only 2s and 3s recorded in the Hunger and Satiety columns of their diary, of consuming fruit and vegetables every day in accordance with the prevailing national guidelines, and of the importance of gradually attaining and maintain physical activity levels of approximately 8,000 to 12,000 steps per day or its equivalent).
- Review participants' practice of the second exercise in Handout 10 (*Aim: Focus on positive aspects of your body*), and explore their reactions to this exercise.

Dissatisfaction with body weight and shape (continued)

Strong dissatisfaction with body image is reviewed in this session, as it is a key factor contributing to disordered eating behavior and consequent weight gain. In this activity, the therapist's aim is to help participants to understand what may contribute to any overwhelming dissatisfaction they may have with their body image. The therapist asks the participants to think about factors that may have contributed to their dissatisfaction with their body image, notably weight and shape. The therapist can ask about any negative influences that media, relatives or friends may have had on the development of any excessive dissatisfaction with weight and shape that participants may feel. It is advised that the therapist emphasize that excessive body weight is not healthy as it can increase the chances of developing some diseases (e.g. diabetes, cardiovascular disease, arthritis), however the focus on a "perfect" body is not healthy, either. To address these issues the therapist could use questions such as:

- Do you remember any situation that made you feel particularly dissatisfied with your weight and/or shape? If yes, what do you think contributed to this feeling? How can you manage a similar situation differently in the future?

- Do you have a particular weight or shape that you would like to achieve? Why would you like to achieve this particular weight or shape? What are the pros and cons of trying to achieve this particular weight or shape? If participants have the aim of achieving an unrealistic or "perfect" weight or shape, the therapist can then ask questions such as: How much success do you feel you have had in achieving this particular weight or shape? How do you feel when you are regularly trying to achieve this goal without success? What can you do about this situation?

Identifying my good points

In this activity, the therapist is to help participants notice their positive characteristics (not necessarily related to weight, shape or eating) in order to improve their self-esteem. This is important because a positive emotional state could be helpful in overcoming disordered eating (13) and could aid with weight loss. In order to introduce this activity to participants, the therapist can ask questions such as the following:

- Do you think that your emotional states affect your eating habits?
- Do you remember any situation when you were feeling sad, anxious or stressed and you were attempting to lose weight?
- If yes, how was this experience?
- Do you think that if you were in a more positive emotional or psychological state that you would have had different results?

It is recommended that after this discussion, the therapist ask participants to start answering the questions in Handout 11 in session, and to finish answering these questions at home. The therapist can explain that this activity helps people to develop a more positive and balanced view of themselves.

Homework

- Participants continue recording / self-monitoring their eating behavior and physical activity.
    - Remind participants to bring their *HAPIFED Diary* (Handout 1) *and HAPIFED summary sheet* (Handout 2) to the next session.
- Participants are asked to finish the exercise of *Identifying my good points* in Handout 11, and to bring this handout to the next session.

Handout 11: Identifying my good points
(Reproduced with thanks from Adult Psychology resources Campbelltown Community Mental Health Sydney South West Area Health Service.)

What do you like about yourself, however small and fleeting?

....

....

....

....

What positive qualities do you possess?

....

....

....

....

What have you achieved in life, however small?

....

....

....

....

What challenges have you overcome?

....

....

....

....

What gifts or talents do you have, however modest?

....

....

....

....

What skills have you acquired?

....

....

....

....

What do other people like or value in you?

....

....

....

What qualities and actions that you value in others do you share?

....

....

....

What aspects of yourself would you appreciate if they were aspects of another person?

....

....

....

What small positives are you discounting?

....

....

....

What are the bad things you are not?

....

....

....

How might another person who cared about you describe you?

....

....

....

....

Look at this list a few days after you have written it. Is there anything you can add to it?

....

....

....

....

Session 13: Body checking, comparison and avoidance
(Stage 2: Core interventions)

Material to be distributed at this session

- Handout 3: *Bi-weekly monitoring worksheet*
- Handout 12: *Self-nurturing activities*

Check Homework

- Review recording / self-monitoring of participants' eating behaviour and physical activity (the therapist reminds participants of the importance of having only 2s and 3s recorded in the Hunger and Satiety columns of their diary, of consuming fruit and vegetables every day in accordance with the prevailing national guidelines, and of the importance of gradually attaining and maintain physical activity levels of approximately 8,000 to 12,000 steps per day or its equivalent).
- Review participants' experiences of the exercise from Handout 11 entitled *Identifying my good points*.

Bi-weekly monitoring of participants' progress with behavioral weight loss therapy

In this activity, the therapist's aims are the following:

- Encourage participants to improve the quality of their recording of eating behavior.
- Assess participants' progress with the program.
- Identify any difficulties or barriers to change, and assist with strategies to resolve these.

The *Bi-weekly monitoring worksheet* (Handout 3) is again distributed to participants and the monitoring is done in session. The therapist should remind participants to transfer their data from the *Bi-weekly monitoring worksheet* to their *HAPIFED summary sheet* (Handout 2), and any emerging patterns should be discussed in order to assist participants in achieving the goals of eating within hunger and satiety levels of ±2 and ±3, achieving adequate intake of fruit and vegetables, and achieving physical activity levels commensurate with long-term successful weight loss (8,000 to 12,000 steps per day or its equivalent).

Body checking, comparison and avoidance

In this activity, the therapist is to help elicit reduction in any excessive body image checking, comparison or avoidance that participants may be engaging in, as these behaviors can contribute to the maintenance of eating disorders (1). It is recommended that the therapist define what is meant by body checking, and state that most people engage in some degree of body checking and comparing their bodies with others, however this may become obsessive and dysfunctional for some individuals. It is appropriate that the therapist emphasizes the following two points: 1) that excessive body checking and comparison with others may reinforce dissatisfaction with body image and trigger disordered eating behavior (e.g. extreme dietary restriction leading to binge eating episodes), and that 2) avoiding seeing and checking one's own body may also be problematic, as never realizing what one actually looks like can maintain body dissatisfaction.

It is suggested that the therapist ask participants about their experiences of body checking, comparing their body with others' bodies and avoidance of seeing their body, and assesses if their behavior seems dysfunctional. If this is the case, the therapist should discuss with participants ways of dealing with body image checking, comparison with others or avoidance that do not trigger dissatisfaction with body image. It is important that the therapist emphasizes that these behaviors are not problematic as long as they occur in moderation and do not trigger strong body image dissatisfaction.

Self-nurturing activities

In this activity, the therapist is to encourage participants to engage in activities that improve their mood in order to reduce their likelihood of engaging in binge eating and compensatory behaviors. In order to start a discussion about this topic, the therapist can ask questions such as:

- Do you think your emotional state influences your eating behavior?
- What are activities you enjoy that usually improve your mood?

Next, the therapist and participants jointly write a list (as long as possible) of the aforementioned pleasurable activities. In order to help generate ideas for the list, the therapist can ask participants what they have successfully done in the past to improve their mood. The therapist can also suggest examples of activities that usually improve people's emotional state, such as going for walk in a park, going to the beach, watching a fun film, meeting friends, and listening to music. After the therapist and participants have written the joint list of pleasurable activities, participants are asked to answer the questions in Handout 12 (*Self-nurturing activities*) about some pleasurable activities that they can do in the near future.

Homework

- Participants continue recording / self-monitoring their eating behavior and physical activity.
    - Remind participants to bring their *HAPIFED Diary* (Handout 1) *and HAPIFED summary sheet* (Handout 2) to the next session.
- In addition to the above, this week the written self-monitoring also includes recording of any excessive weight or shape checking or avoidance (this information can be written in the Context column).
- Participants are encouraged to practice self-nurturing activities, or to get organized to start practicing these activities.

Handout 12: Self-nurturing activities

What types of pleasurable activities can you do in the near future?

....

....

....

....

....

....

When is the best time for you to engage in these activities (e.g. what day of the week or time of the day)?

....

....

....

....

....

....

What do you need to organize in order to start these activities (e.g. talk with friends, buy a set of headphones to listen to music)?

....

....

....

....

....

....

.

Session 14: Physical activity education (continued)
(Stage 2: Core interventions)

Material to be distributed at this session

- Exercise physiology education material

Check Homework

- Review recording / self-monitoring of participants' eating behaviour and physical activity (the therapist reminds participants of the importance of having only 2s and 3s recorded in the Hunger and Satiety columns of their diary, of consuming fruit and vegetables every day in accordance with the prevailing national guidelines, and of the importance of gradually attaining and maintain physical activity levels of approximately 8,000 to 12,000 steps per day or its equivalent).
- Review participants' engagement with self-nurturing activities or organization to start practicing these activities.

Physical activity education with exercise physiologist (continued)

Suggested topics:

- Review of key points from Session 5.
- Review of any changes in participants' physical activity, drawing on data that has been collected into the *HAPIFED Diary* (Handout 1), monitored with the *Bi-weekly monitoring worksheets* (Handout 3) and collated into the *HAPIFED summary sheet* (Handout 2).
- Address participants' questions.

Homework

- Participants continue recording / self-monitoring their eating behavior and physical activity.
    - Remind participants to bring their *HAPIFED Diary* (Handout 1) *and HAPIFED summary sheet* (Handout 2) to the next session.

Session 15: Nutritional education
(Stage 2: Core interventions)

Material to be distributed at this session

- Nutritional education material
- Handout 3: *Bi-weekly monitoring worksheet*

Check Homework

- Review recording / self-monitoring of participants' eating behaviour and physical activity (the therapist reminds participants of the importance of having only 2s and 3s recorded in the Hunger and Satiety columns of their diary, of consuming fruit and vegetables every day in accordance with the prevailing national guidelines, and of the importance of gradually attaining and maintain physical activity levels of approximately 8,000 to 12,000 steps per day or its equivalent).
- Review participants' attitude towards physical activity or exercise since last session, and whether they noted any changes in their physical activity or exercise habits.

Bi-weekly monitoring of participants' progress with behavioral weight loss therapy

In this activity, the therapist's aims are the following:

- Encourage participants to improve the quality of their recording of eating behavior.
- Assess participants' progress with the program.
- Identify any difficulties or barriers to change, and assist with strategies to resolve these.

The *Bi-weekly monitoring worksheet* (Handout 3) is again distributed to participants and the monitoring is done in session. The therapist should remind participants to transfer their data from the *Bi-weekly monitoring worksheet* to their *HAPIFED summary sheet* (Handout 2), and any emerging patterns should be discussed in order to assist participants in achieving the goals of eating within hunger and satiety levels of ±2 and ±3, achieving adequate intake of fruit and vegetables, and achieving physical activity levels commensurate with long-term successful weight loss (8,000 to 12,000 steps per day or its equivalent).

Nutritional education with dietitian (continued)

Suggested topics:

- Review of key points from Session 4, remembering to keep a focus on the types and proportions of different foods that are required for health, not on portion sizes or kilojoule counts that are required for weight loss. This program is designed to reduce energy intake relative to energy expenditure implicitly rather than explicitly, by encouraging the consumption of healthy foods according to appetite, by encouraging physical activity, and via regular use of the *HAPIFED Diary* (Handout 1).
- Review any changes in food and beverage selections and eating behavior that participants may have noticed since Session 4.
- Address any questions that participants may have.

Homework

- Participants continue recording / self-monitoring their eating behavior and physical activity.
    - Remind participants to bring their *HAPIFED Diary* (Handout 1) *and HAPIFED summary sheet* (Handout 2) to the next session.

Session 16: Increasing self-confidence
(Stage 2: Core interventions)

Material to be distributed at this session

- Handout 5: *Proactive problem solving sheet*
- Handout 13: *Fifteen steps to a more confident you*

Check Homework

- Review recording / self-monitoring of participants' eating behaviour and physical activity (the therapist reminds participants of the importance of having only 2s and 3s recorded in the Hunger and Satiety columns of their diary, of consuming fruit and vegetables every day in accordance with the prevailing national guidelines, and of the importance of gradually attaining and maintain physical activity levels of approximately 8,000 to 12,000 steps per day or its equivalent).

Creating an helpful home environment

In this activity, the therapist is to explain that having a home environment that facilitates healthy eating and healthy exercise will increase the chances of participants' maintenance of healthy eating and exercise habits. It is recommended that the therapist stimulates a discussion in regards to how their current home environment influences participants' eating and exercise habits. The therapist can give examples of a home environment that is not helpful when a big container full of assorted chocolates is exposed in the living room and this triggers overeating of chocolate. Next, the therapist can give an example of a helpful home environment when plenty of fresh fruit is available and easily visible or when some delicious salad is prepared in advance and placed in the freezer for the next meals. Ultimately, the aim of this activity is to engage participants in brainstorming solutions to keep a home environment that will facilitate their engagement in healthy eating and healthy exercise habits. As homework, it is advised that the therapist asks the participants to engage in proactive problem solving (using Handout 5) with the aim of making changes in their home environment that can facilitate healthy eating and healthy exercising.

Increasing self-confidence

In this activity, the therapist is to aid participants with increasing their self-confidence. It is recommended that the therapist explain that self-confidence is important because it will help participants to reduce disordered eating and achieve weight management goals. For this activity it is suggested that Handout 13, entitled *Fifteen steps to a more confident you*, is read in group and the therapist stimulate a discussion around the importance of increasing self-confidence for improving eating behavior, as well as how to increase self-confidence. In order to engage participants in this discussion, the therapist can ask participants if any specific parts of the text come to their attention as meaningful to them.

Homework

- Participants continue recording / self-monitoring their eating behavior and physical activity.
    - Remind participants to bring their *HAPIFED Diary* (Handout 1) *and HAPIFED summary sheet* (Handout 2) to the next session.
- Participants are asked to engage in proactive problem solving (using Handout 5) with the aim of making changes in their home environment that can facilitate healthy eating and healthy exercising, and to bring this handout to the next session.
- Participants are asked to read Handout 13 on *Fifteen steps to a more confident you* again at home, and try to implement the outlined steps.

Handout 13: Fifteen steps to a more confident you

(Reproduced with thanks from Adult Psychology resources Campbelltown Community Mental Health Sydney South West Area Health Service.)

1. Recognize your strengths and weaknesses and set your goals accordingly.
2. Decide what you value, what you believe in, what you realistically would like your life to be like. Take inventory of your library of stored scripts and bring them up to date, in line with the psychological space you are in now, so they will serve you where you are headed.
3. Determine what your roots are. By examining your past, seek out the lines of continuity and the decisions that have brought you to your present place. Try to understand and forgive those who have hurt you and not helped when they could have. Forgive yourself for mistakes, sins, failures, and past embarrassments. Permanently bury all negative self remembrances after you have sifted out any constructive value they may provide. The bad past lives on in your memory only as long as you let it be a tenant. Prepare an eviction notice immediately. Give the room to memories of your past successes, however minor.
4. Guilt and shame have limited personal value in shaping your behavior towards positive goals. Don't allow yourself to indulge in them.
5. Look for the causes of your behavior in physical, social, economic, and political aspects of your current situation and not in personality defects in you.
6. Remind yourself that there are alternative views to every event. 'Reality' is never more than shared agreements among people to call it the same way rather than as each one separately sees it. This enables you to be more tolerant in your interpretation of others' intentions and more generous in dismissing what might appear to be rejections or put-downs of you.
7. Never say bad things about yourself; especially, never attribute to yourself irreversible negative traits, like 'stupid', 'ugly', 'uncreative' or 'a failure'.
8. Don't allow others to criticize you as a person; it is your specific actions that are open for evaluation and available for improvement – accept such constructive feedback graciously if it will help you.
9. Remember that sometimes failure and disappointment are blessings in disguise, telling you the goals were not right for you, the effort was not worth it, and a bigger letdown later on may be avoided.
10. Do not tolerate people, jobs, and situations that make you feel inadequate. If you can't change them or yourself enough to make you feel more worthwhile, walk on out, or pass them by. Life is too short to waste time on downers.
11. Give yourself the time to relax, to meditate, to listen to yourself, to enjoy hobbies and activities you can do alone. In this way, you can get in touch with yourself.
12. Practice being a social animal. Enjoy feeling the energy that other people transmit, the unique qualities and range of variability of our brothers and sisters. Imagine what their fears and insecurities might be and how you could help them. Decide what you need from them and what you have to give. Then, let them know that you are ready and open to sharing.
13. Stop being so overprotective about your ego; it is tougher and more resilient that you imagine. It bruises but never breaks. Better it should get hurt occasionally from an emotional commitment that didn't work out as planned, than get numbed from the emotional insulation of playing it too cool.
14. Develop long-range goals in life, with highly specific short-range sub goals. Develop realistic means to achieve these sub goals. Evaluate your progress regularly and be the first to pat yourself on the back or whisper a word of praise in your ear. You don't have to worry about being unduly modest if no one else hears you boasting.
15. You are not an object to which bad things just happen, a passive nonentity hoping, like a garden slug, to avoid being stepped on. You are the culmination of millions of years of evolution of our species and of your parents' dreams. You are a unique individual who, as an active actor in life's drama, can make things happen. You can change the direction of your entire life any time you choose to do so. With confidence in yourself, obstacles turn into challenges and challenges into accomplishments. Shyness then recedes, because, instead of always preparing for and worrying about how you will live out your life, you forget yourself as you become absorbed in the living of it.

Session 17: Relaxation / Barriers to change
(Stage 2: Core interventions)

Material to be distributed at this session

- Handout 3: *Bi-weekly monitoring worksheet*
- Handout 5: *Proactive problem solving sheet*
- Handout 14: *Slow breathing*

Check Homework

- Review recording / self-monitoring of participants' eating behaviour and physical activity (the therapist reminds participants of the importance of having only 2s and 3s recorded in the Hunger and Satiety columns of their diary, of consuming fruit and vegetables every day in accordance with the prevailing national guidelines, and of the importance of gradually attaining and maintain physical activity levels of approximately 8,000 to 12,000 steps per day or its equivalent).
- Review participants' proactive problem solving (using Handout 5), with the aim of making changes in their home environment that can facilitate healthy eating and healthy exercising.
- The therapist discusses with participants their experiences of re-reading at home and implementing any of the steps outlined in Handout 13, entitled *Fifteen steps to a more confident you*.

Slow breathing

This activity in *Slow breathing* (Handout 14) aims to reduce participants' stress. In this activity, the therapist is to explain that relaxation exercises provide participants with a valuable skill that they can use in situations of stress. In addition, the therapist can discuss with participants that as a consequence of stress reduction, they may be less likely to use eating as a means of stress reduction. It is recommended that the therapist provide participants with in-session practice of a relaxation exercise, as per the instructions in Handout 15.

Bi-weekly monitoring of participants' progress with behavioral weight loss therapy

At this activity, the therapist aims are the following:
- Encourage participants to improve the quality of their eating behavior recording.
- Assess participants' progress with the program.
- Identify any difficulties or barriers to change and assist with strategies to resolve these.

The *Bi-weekly monitoring worksheet* (Handout 3) is again introduced to participants and the monitoring is done in session. The therapist should remind participants to transfer their data from the *Bi-weekly monitoring worksheet* to their *HAPIFED summary sheet* (Handout 2), and any emerging patterns should be discussed in order to assist participants in achieving the goals of eating within hunger and satiety levels of ±2 and ±3, achieving adequate intake of fruit and vegetables, and achieving physical activity levels commensurate with long-term successful weight loss (8,000 to 12,000 steps per day or its equivalent).

Barriers to change

In this activity, the therapist is to assess participants' attitudes towards, and engagement with, the HAPIFED Program. It is recommended that the therapist ask participants to "brainstorm" difficulties they face with any aspects of the program, and any barriers to following aspects of the program are discussed in the group, with the aim of exploring potential solutions. It is important that the therapist mentions common reasons for the therapy not being successful for some people (1), such as intermittent attendance (for example, due to competing commitments that interfere with the program schedule), not completing the *HAPIFED Diary*, not reading the recommended texts, ambivalence to change, difficulty with planning, low mood, low self-efficacy, perfectionism, substance abuse, dislike of the program or therapist, and/or inappropriate interventions by the therapist. These barriers should be explored and discussed with participants. If necessary, the *HAPIFED formulation of loss of control over eating and weight gain* can be revised individually with each participant, as it may provide additional information about each participant's particular barriers to change, if any. After identification of barriers to change that participants may be experiencing with the HAPIFED Program, they are asked to engage in proactive problem solving (Handout 5) at home or – if there is enough time – in session with facilitation by the therapist.

Homework

- Participants continue recording / self-monitoring their eating behavior and physical activity.
    - Remind participants to bring their *HAPIFED Diary* (Handout 1) *and HAPIFED summary sheet* (Handout 2) to the next session.
- Participants are asked to engage in proactive problem solving (using Handout 5) regarding any barriers that they may be facing in successfully engaging with the HAPIFED Program, and to bring this handout to the next session.
- Participants are invited to practice *Slow breathing* (Handout 14)

Handout 14: Slow breathing

- Make yourself comfortable in a chair or lying down.
- [At home: if you like you can put on some music (calming music is better)].
- Close your eyes.
- Now place one hand on your upper belly, and breathe deeply and slowly from the upper belly and diaphragm. Try to make your belly expand as much as possible as you breathe in. You will know that you are doing it correctly (that is, breathing from your upper belly and diaphragm) when you feel your belly getting bigger as you breathe in (not your chest).
- Inhale deeply and slowly counting 4 seconds, hold for 4 seconds, and then exhale deeply and slowly counting 4 seconds.
- Continue for approximately 5 minutes.
    - Note – saying or thinking "one thousand" takes approximately one second in time.

30 Group Session Program Manual © da Luz, Swinbourne, Hay, Touyz, Palavras, Claudino, Sainsbury 2017

Session 18: Progressive muscle relaxation / Barriers to change (continued)
(Stage 2: Core interventions)

Material to be distributed at this session

- Handout 5: *Proactive problem solving sheet*
- Handout 14: *Slow breathing*
- Handout 15: *Progressive muscle relaxation*

Check Homework

- Review recording / self-monitoring of participants' eating behaviour and physical activity (the therapist reminds participants of the importance of having only 2s and 3s recorded in the Hunger and Satiety columns of their diary, of consuming fruit and vegetables every day in accordance with the prevailing national guidelines, and of the importance of gradually attaining and maintain physical activity levels of approximately 8,000 to 12,000 steps per day or its equivalent).
- Review participants' engagement in proactive problem solving (Handout 5) regarding any barriers to change they may experience in engagement with the HAPIFED Program, and any solutions that they generated.
- Review participants' practice of the relaxation exercise entitled *Slow breathing* (Handout 14).

Progressive muscle relaxation

This relaxation exercise (Handout 15) aims to reduce participants' stress. It is important to do this activity because stress can often trigger disordered eating (14, 15), and if participants learn how to manage stress in a healthy manner they may be less likely to engage in disordered eating. In this activity, the therapist is to explain that progressive muscle relaxation is a valuable relaxation exercise that they can use in situations of stress, especially for people who experience muscle tension associated with stress. This exercise enables people to notice the difference between tension and relaxation in each of their muscle groups, and to scan their bodies for residual tension. It is recommended that the therapist provide participants with an in session practice of progressive muscle relaxation, in accordance with the instructions in Handout 15.

Note: if participants have medical conditions (e.g. chronic pain or high blood pressure), they should consult a doctor before doing this exercise.

<u>Barriers to change</u> (continued)

In this activity, the therapist is to continue helping participants to identify any barriers to change they may be experiencing, and to help them find potential solutions. The following steps are suggested:

- The therapist asks participants to reflect on the major barrier to change that they experience with the program.
- The therapist then asks each participant, one at a time, if they would feel comfortable to share his/her major barrier to change with the group.
- The therapist next seeks input from other members of the group as to how they might deal with this barrier to change, if they were experiencing it themselves. It is important that participants do not sound like they are telling other participants what they should do; only what they themselves think they might do if they were experiencing the same barrier to change. The therapist can prompt: 1) review of previous sessions covering potential strategies for addressing barriers to change, and 2) implementing structured problem solving with the group.
- This process is repeated until all participants have had an opportunity to share their individual barrier to change with the group, if desired, and are supported by potential solutions to consider from other participants.

Note: the aim of this activity is to utilize the group dynamic in order to help participants to cope with their individual barriers to change, and to promote engagement with the therapy program. The participants may have identified new barriers to change since the previous session, or they may identify new barriers to change in this session, after thinking about it further and/or hearing about other participants' difficulties. Similarly as in Session 15, after the identification of barriers to change that participants may be experiencing with the HAPIFED Program, they are invited to engage in proactive problem solving at home (Handout 15).

<u>Homework</u>

- Participants continue recording / self-monitoring their eating behavior and physical activity.
  - Remind participants to bring their *HAPIFED Diary* (Handout 1) *and HAPIFED summary sheet* (Handout 2) to the next session.
- Participants are invited to practice *progressive muscle relaxation* at home (Handout 15).
- Participants are asked to engage in proactive problem solving (using Handout 5) regarding any barriers to change that they may face with engaging in the HAPIFED Program, and to bring this handout to the next session.

Handout 15: Progressive muscle relaxation

[At Home]: set aside a period of 20 minutes or more when you will not be disturbed: this is <u>your</u> time. Make yourself comfortable in a chair or lying down. [At home]: If you like, put on some calming music.

Begin with *Slow breathing* (Handout 14).

After about 5 minutes of slow breathing, practice tensing and relaxing an isolated part of your body by closing your hand into a fist and tensing the muscles – hold for 5 seconds – then relax. Notice the difference between the feeling of tension and relaxation.

Let's begin…

Tense your toes, hold them tightly for 5 seconds, and then relax for 5 seconds. Notice the difference between the feeling of tension and relaxation.

Now tense the muscles in your feet (you can do this by pulling your toes upwards) for 5 seconds, and relax for 5 seconds. Again, notice the difference between the feeling of tension and relaxation.

Continue this same pattern for your calf muscles, thigh muscles, hip muscles, hands (as fists), forearms, upper arms, neck, and shoulders. Continue onwards by tensing and relaxing the muscles in your head, forehead (scowl), face (frown), chest, and abdominal muscles.

Finally, tense your whole body at once and hold for 5 seconds, then relax. Notice the difference between the feeling of tension and relaxation.

If you notice any areas of residual tension, try to relax those areas by tensing and then relaxing.

At the end of this exercise, you will likely feel much more relaxed than when you started.

You may like to focus on a pleasurable memory or image.

Practice this regularly, once or more a day is ideal. When the relaxation is thoroughly practiced, you will find that you can achieve the same degree of relaxation in a much shorter time, but this takes a lot of practice…

Session 19: Personalization of the formulation
(Stage 2: Core interventions)

<u>Material to be distributed at this session</u>

- Handout 1: *The HAPIFED formulation of loss of control over eating and weight gain*
- Handout 3: *Bi-weekly monitoring worksheet*
- Handout 5: *Proactive problem solving sheet*
- Handout 16: *Personalizing the HAPIFED formulation of loss of control over eating and weight gain*

<u>Check Homework</u>

- Review recording / self-monitoring of participants' eating behaviour and physical activity (the therapist reminds participants of the importance of having only 2s and 3s recorded in the Hunger and Satiety columns of their diary, of consuming fruit and vegetables every day in accordance with the prevailing national guidelines, and of the importance of gradually attaining and maintain physical activity levels of approximately 8,000 to 12,000 steps per day or its equivalent).
- Review participants' practice of progressive muscle relaxation.
- Review participants' engagement in proactive problem solving (Handout 5) regarding any barriers to change that they may face towards engagement with the HAPIFED Program.

<u>Bi-weekly monitoring of participants' progress with behavioral weight loss therapy</u>

In this activity, the therapist's aims are the following:

- Encourage participants to improve the quality of their eating behavior recording.
- Assess participants' progress with the program.
- Identify any difficulties or barriers to change and assist with strategies to resolve these.

The *Bi-weekly monitoring worksheet* (Handout 3) is again introduced to participants and the monitoring is done in session. The therapist should remind participants to transfer their data from the *Bi-weekly monitoring worksheet* to their *HAPIFED summary sheet* (Handout 2), and any emerging patterns should be discussed in order to assist participants in achieving the goals of eating within hunger and satiety levels of ±2 and ±3, achieving adequate intake of fruit and vegetables, and achieving physical activity levels commensurate with long-term successful weight loss (8,000 to 12,000 steps per day or its equivalent).

30 Group Session Program Manual © da Luz, Swinbourne, Hay, Touyz, Palavras, Claudino, Sainsbury 2017

Personalization of the HAPIFED formulation of loss of control over eating and weight gain

In this activity, the therapist is to help participants understand in greater detail the factors that contribute to the maintenance of their problem with eating and weight. It is recommended that the therapist again provide Handout 16 to participants and emphasize that this generic *HAPIFED formulation of loss of control over eating and weight gain* aids a general understanding of factors that commonly contribute to the maintenance of difficulties that people experience with eating and weight. However, specific personal information needs to be added to this formulation in order to develop a "map" of one's personal difficulty with eating and weight. It is advised that the therapist inform participants that after developing a personal "map" of their problems with eating and weight, it will likely be much easier for them to find their "way out" of their difficulties. Next, the therapist can ask participants to add their personal information to the formulation in order to develop the clearest "map" possible of their problems with eating and weight. After participants have finished personalizing their *HAPIFED formulation of loss of control over eating and weight gain*, the therapist can invite participants to engage in proactive problem solving (Handout 5) with a problem that they identified during personalization of the formulation.

Homework

- Participants continue recording / self-monitoring their eating behavior and physical activity.
    - Remind participants to bring their *HAPIFED Diary* (Handout 1) *and HAPIFED summary sheet* (Handout 2) to the next session.
- Participants are asked to review their *Personalization of the HAPIFED formulation of loss of control over eating and weight gain* (Handout 16), and to add further personal information to it, if they feel this is necessary.
    - Remind participants to bring their *Personalization of the HAPIFED formulation of loss of control over eating and weight gain* (Handout 16) to the next session.

Handout 16: Personalizing the HAPIFED formulation of loss of control over eating and weight gain

Please add your personal information to the blank spaces inside the boxes of the HAPIFED formulation on the following page. There is no need to share your personal information with other participants in the group or with the therapist, but feel free to do so if you wish. Adding your personal information to the formulation will help you to gain a clearer understanding of what may be maintaining your difficulties with eating and weight. The questions below may help you to see the type of information that can be added to the formulation.

Strong dissatisfaction with body image, notably weight and shape
Does this apply to you? If yes, can you describe the intensity of your dissatisfaction with your body image, notably weight and shape? Are there specific things that trigger dissatisfaction with your body image, notably, weight and shape?

Disordered eating behaviors: severe and unhealthy dietary restriction; purging (vomiting, use of laxatives or diuretics); excessive and compulsive exercise
Do you engage in any of these types of behavior? If yes, which ones and how often? What consequences of these behaviors do you experience?

Emotional states: sadness, irritation, frustration, anxiety, excitement, stress…
Do you think that your emotional states change your eating behavior? If yes, how do your emotional states change your eating behavior (e.g. eating more, eating less…)? How often do your emotional states change your eating behavior?

Difficulty tolerating the desire to eat (when experiencing no real hunger)
Does this apply to you? If yes, why do you think this happens?

Loss of control over eating and consequent weight gain
What type of foods or beverages do you usually consume when you experience loss of control over eating? How much do you usually consume in these situations? How do you feel physically and emotionally after eating in this way?

Personalizing the HAPIFED formulation of loss of control over eating and weight gain

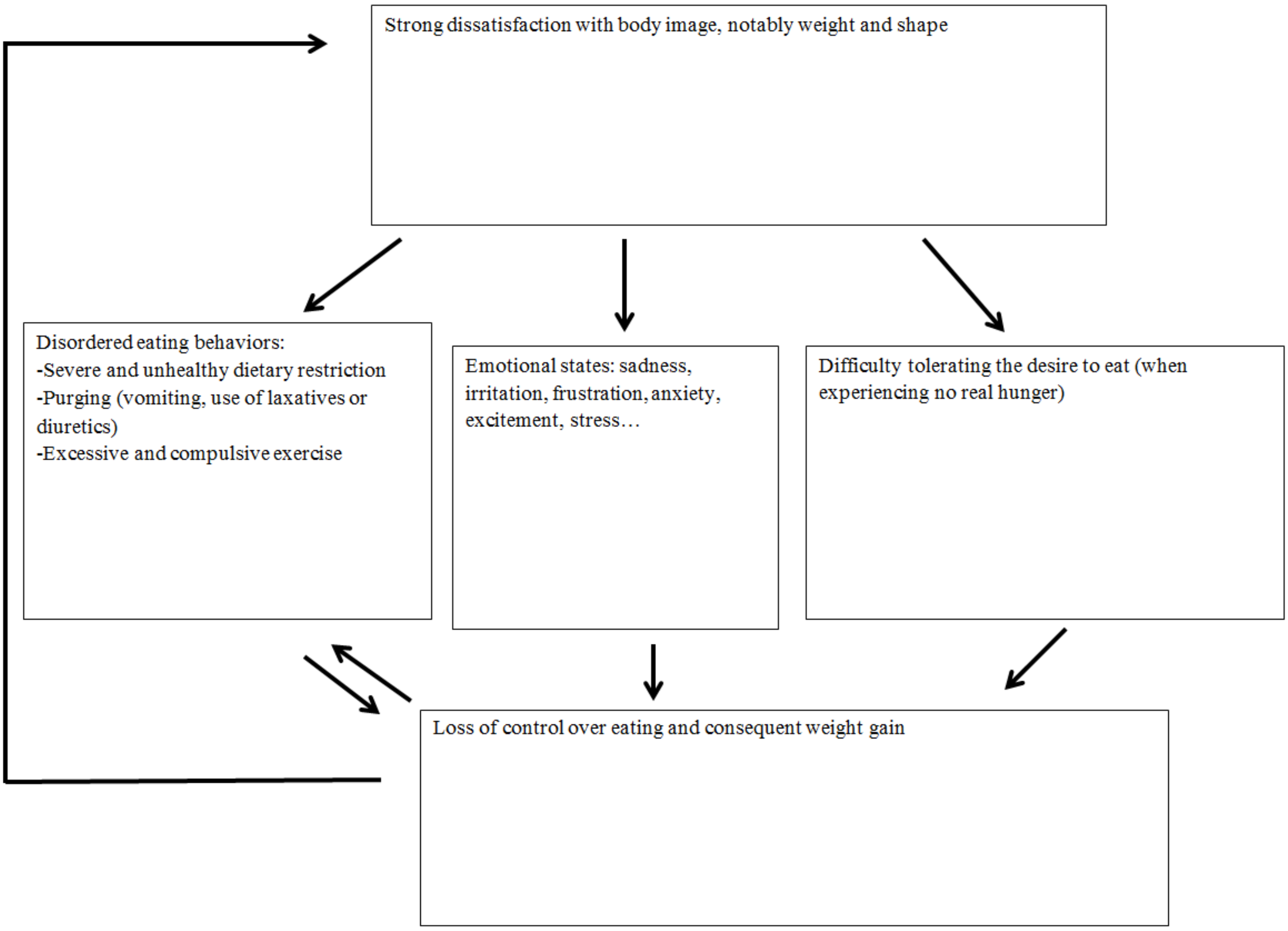

Session 20: Unhelpful thinking styles

(Stage 2: Core interventions)

Material to be distributed at this session

- Two copies of Handout 17: *Cognitive distortions questionnaire (CD-Quest)*

Check Homework

- Review recording / self-monitoring of participants' eating behaviour and physical activity (the therapist reminds participants of the importance of having only 2s and 3s recorded in the Hunger and Satiety columns of their diary, of consuming fruit and vegetables every day in accordance with the prevailing national guidelines, and of the importance of gradually attaining and maintain physical activity levels of approximately 8,000 to 12,000 steps per day or its equivalent).
- Review any additional information that participants may have added to their *Personalized HAPIFED formulation of loss of control over eating and weight gain* (Handout 16), and any insights that they may have gained from that exercise.

Unhelpful thinking styles and disordered eating behaviors

In this activity, the therapist's aim is to discuss with participants the following two points: 1) the influence of their thoughts and beliefs on their eating behaviors, and 2) the importance of cultivating helpful thinking styles in order to overcome disordered eating. It is recommended that the therapist explain that it is important to be aware of one's thoughts and beliefs, because some people may engage in an eating disorder mindset (1) that keeps them engaged in unhealthy eating behaviors. The therapist can explain to participants that this activity requires participants to attempt to increase awareness of any personal thought processes (cognitions) that may trigger or maintain disordered eating; and that their ultimate goal should be to replace any dysfunctional thoughts with more functional thoughts. In order to facilitate participants' understanding of the meaning of "unhelpful thoughts and beliefs", the following examples of cognitive distortions, which are common amongst individuals with disordered eating (4), can be mentioned by the therapist:

- Using body image, notably weight and/or shape, as the main means of self-evaluation, disregarding other important personal qualities (e.g. intelligence, competency, being a responsible parent).
- Thinking that everyone is constantly paying attention to and judging a person according to weight or shape.
- Thinking in terms of "good foods" (e.g. vegetables) and "bad foods" (e.g. carbohydrates, fat, sugar), and that people should eat only "good foods" and never eat "bad foods". Disregarding the fact that eating any food in moderation will not lead to weight gain.
- Thinking that if some "fattening food" is consumed, that it is a sign of being out of control, and that there is no point trying to continue with efforts to eat healthfully or moderately.
- Holding the perception of oneself and/or the world in terms of black and white terms, such as acceptable or unacceptable, worthwhile or worthless, good or bad (all or nothing thinking); not considering the existence of a continuum of possibilities.
- Overestimation of the benefits of being under a certain weight. Thinking that all of a person's desires in life will be accomplished by achieving a certain lower weight.

- Thinking that every kilojoule can be removed from the body without consequence by vomiting, misuse of laxatives/diuretics, extreme exercise or severe dietary restriction.

Exercise on cognitive distortions

In this activity, the therapist is to ask participants to answer the *Cognitive distortions questionnaire (CD-Quest*, Handout 17) in session. The aim of this activity is to help participants to notice how much and what type of cognitive distortions they tend to engage in, thereby helping them to be more aware of any unhelpful thinking styles that may contribute to disordered eating. It is important that the therapist emphasizes the importance of the participants' writing their own examples of cognitive distortions on the *Cognitive distortions questionnaire*; especially those cognitive distortions related to their eating behavior.

Homework

- Participants continue recording / self-monitoring their eating behavior and physical activity.
    - Remind participants to bring their *HAPIFED Diary* (Handout 1) *and HAPIFED summary sheet* (Handout 2) to the next session.
- Participants are asked to answer the *Cognitive distortions questionnaire (CD-Quest)* again at home, using a fresh copy of Handout 17, in order to help increase their awareness of distorted or dysfunctional thinking styles.
    - Remind participants to bring their *Cognitive distortions questionnaire (CD-Quest)* to the next session.

Handout 17: Cognitive distortions questionnaire (CD-Quest)
(Reproduced from De Oliveira I. R. *Trial-Based Cognitive Therapy: A Manual for Clinicians*. New York: Routledge; 2014, p. 28-35, with permission from Professor Irismar Oliveira (16))

Copyright (c) 2010
Irismar Reis de Oliveira, MD, PhD
Department of Neurosciences and Mental Health
Federal University of Bahia, Brazil

All of us have thousands of thoughts a day. These thoughts are words, sentences and images that pop into our heads as we are doing things. Many of these thoughts are accurate, but many are distorted. This is why they are called cognitive errors or cognitive distortions.

For example, Paul is a competent journalist who had his ten-page work assessed by John, the editor of an important local newspaper. John amended one paragraph and made a few other suggestions of minor importance. Although John approved Paul's text, Paul became anxious and found himself thinking: "This work is not good at all. If it were good, John wouldn't have made any correction."

For Paul, either the work is good or it is bad. This kind of thinking error is sometimes called dichotomous thinking. As this thought returned to Paul's mind several times from Friday to Sunday (3 days), and Paul believed it at least 75%, he made a circle around number 4 in the fourth column of the grid below.

1. **Dichotomous thinking (also called all-or-nothing, black and white, or polarized thinking):** I view a situation, a person or an event in "either-or" terms, fitting them into only two extreme categories instead of on a continuum.
EXAMPLES: "I made a mistake, therefore my performance was a failure". "I ate more than I planned, so I blew my diet completely".
Paul's example: "This work is not good at all. If it were good, John wouldn't have made any correction".

**Frequency:**<br>**Intensity:**	**No** (It did not occur)	**Occasional** (1-2 days during the past week)	**Much of the time** (3-5 days during the past week)	**Almost all of the time** (6-7 days during the past week)
**I believed it...**	0			
A little (Up to 30%)		1	2	3
Much (31% to 70%)		2	3	4
Very much (More than 70%)		3	4	5

Please, turn the page and assess your own thinking style.

Cognitive Distortions Questionnaire (CD-Quest)

Irismar Reis de Oliveira, MD, PhD

Name: .... Date: ....

Please, make a circle around the number corresponding to each option below, indicating cognitive errors or distortions that you have made <u>during this past week</u>. When assessing each cognitive distortion, please, indicate <u>how much</u> you believed it in the exact moment it occurred (not how much you believe it now), and <u>how often</u> it occurred during this past week. Please, give your own examples in the items you mark 3 or more.

DURING THIS PAST WEEK, I FOUND MYSELF THINKING THIS WAY:

1. **Dichotomous thinking (also called all-or-nothing, black and white, or polarized thinking):** I view a situation, a person or an event in "either-or" terms, fitting them into only two extreme categories instead of on a continuum.
EXAMPLES: "I made a mistake, therefore my performance was a failure". "I ate more than I planned, so I blew my diet completely"

My example:………

**Frequency:**<br>**Intensity:**	**No** (It did not occur)	**Occasional** (1-2 days during the past week)	**Much of the time** (3-5 days during the past week)	**Almost all of the time** (6-7 days during the past week)
**I believed it...**	0			
A little (Up to 30%)		1	2	3
Much (31% to 70%)		2	3	4
Very much (More than 70%)		3	4	5

2. **Fortune telling (also called catastrophizing):** I predict the future in negative terms and believe that what will happen will be so awful that I will not be able to stand it.
EXAMPLES: "I will fail and this will be unbearable." "I'll be so upset that I won't be able to concentrate for the exam."

My example:………

**Frequency:**<br>**Intensity:**	**No** (It did not occur)	**Occasional** (1-2 days during the past week)	**Much of the time** (3-5 days during the past week)	**Almost all of the time** (6-7 days during the past week)
**I believed it...**	0			
A little (Up to 30%)		1	2	3
Much (31% to 70%)		2	3	4
Very much (More than 70%)		3	4	5

3. **Discounting the positive:** I disqualify positive experiences or events insisting that they do not count.
EXAMPLES: "I passed the exam, but I was just lucky." "Going to college is not a big deal, anyone can do it."

My example:………

**Frequency:**<br>**Intensity:**	**No** (It did not occur)	**Occasional** (1-2 days during the past week)	**Much of the time** (3-5 days during the past week)	**Almost all of the time** (6-7 days during the past week)
**I believed it...**	0			
A little (Up to 30%)		1	2	3
Much (31% to 70%)		2	3	4
Very much (More than 70%)		3	4	5

30 Group Session Program Manual © da Luz, Swinbourne, Hay, Touyz, Palavras, Claudino, Sainsbury 2017

4. **Emotional reasoning:** I believe my emotions reflect reality and let them guide my attitudes and judgments.
EXAMPLES: "I feel she loves me, so it must be true." "I am terrified of airplanes, so flying must be dangerous." "My feelings tell me I should not believe him."

My example:………

**Frequency:** **Intensity:**	**No** (It did not occur)	**Occasional** (1-2 days during the past week)	**Much of the time** (3-5 days during the past week)	**Almost all of the time** (6-7 days during the past week)
**I believed it...**	0			
A little (Up to 30%)		1	2	3
Much (31% to 70%)		2	3	4
Very much (More than 70%)		3	4	5

5**. Labeling*:*** I put a fixed, global label, usually negative, on myself or others.
EXAMPLES: "I'm a loser." "He's a rotten person." "She's a complete jerk."

My example:………

**Frequency:** **Intensity:**	**No** (It did not occur)	**Occasional** (1-2 days during the past week)	**Much of the time** (3-5 days during the past week)	**Almost all of the time** (6-7 days during the past week)
**I believed it...**	0			
A little (Up to 30%)		1	2	3
Much (31% to 70%)		2	3	4
Very much (More than 70%)		3	4	5

6. **Magnification/minimization:** I evaluate myself, others, and situations placing greater importance on the negatives and/or placing much less importance on the positives.
EXAMPLES: "I got a B. This proves how bad my performance was." "I got an A. It means the test was too easy."

My example:………

**Frequency:** **Intensity:**	**No** (It did not occur)	**Occasional** (1-2 days during the past week)	**Much of the time** (3-5 days during the past week)	**Almost all of the time** (6-7 days during the past week)
**I believed it...**	0			
A little (Up to 30%)		1	2	3
Much (31% to 70%)		2	3	4
Very much (More than 70%)		3	4	5

7. **Selective abstraction (also called mental filter and tunnel vision):** I pay attention to one or a few details and fail to see the whole picture.
EXAMPLES: "Michael pointed out an error in my work. So, I can be fired" (not considering Michael's overall positive feedback). "I can't forget that a small peace of information I gave during my presentation was wrong" (not considering its success and the audience's great applause).

My example:………

**Frequency:** **Intensity:**	**No** (It did not occur)	**Occasional** (1-2 days during the past week)	**Much of the time** (3-5 days during the past week)	**Almost all of the time** (6-7 days during the past week)
**I believed it...**	0			
A little (Up to 30%)		1	2	3
Much (31% to 70%)		2	3	4
Very much (More than 70%)		3	4	5

8. **Mind reading:** I believe that I know the thoughts or intentions of others (or that they know my thoughts or intentions) without having sufficient evidence.
EXAMPLES: "He's thinking that I failed". "She thought I didn't know the project." "He knows I do not like to be touched this way."

My example:………

**Frequency:** **Intensity:**	**No** (It did not occur)	**Occasional (**1-2 days during the past week)	**Much of the time (**3-5 days during the past week)	**Almost all of the time (**6-7 days during the past week)
**I believed it...**	0			
A little (Up to 30%)		1	2	3
Much (31% to 70%)		2	3	4
Very much (More than 70%)		3	4	5

9. **Overgeneralization:** I take isolated negative cases and generalize them, transforming them in a never-ending pattern, by repeatedly using words such as "always", "never", "ever", "whole", "entire", etc.
EXAMPLES: "It was raining this morning, which means it will rain during the whole weekend." "What a bad luck! I missed the plane, so this will interfere in my entire vacation". "My headache will never stop".

My example:………

**Frequency:** **Intensity:**	**No** (It did not occur)	**Occasional (**1-2 days during the past week)	**Much of the time (**3-5 days during the past week)	**Almost all of the time (**6-7 days during the past week)
**I believed it...**	0			
A little (Up to 30%)		1	2	3
Much (31% to 70%)		2	3	4
Very much (More than 70%)		3	4	5

10. **Personalization:** I assume that others' behaviors and external events concern (or are directed to) myself without considering other plausible explanations.
EXAMPLES: "I thought I was disrespected because the cashier did not say thank you to me" (not considering that the cashier did not say thank you to anyone). "My husband left me because I was a bad wife" (not considering that she was his fourth wife).

My example:………

**Frequency:** **Intensity:**	**No** (It did not occur)	**Occasional (**1-2 days during the past week)	**Much of the time (**3-5 days during the past week)	**Almost all of the time (**6-7 days during the past week)
**I believed it...**	0			
A little (Up to 30%)		1	2	3
Much (31% to 70%)		2	3	4
Very much (More than 70%)		3	4	5

11. **Should statements (also "musts", "oughts", "have tos"):** I tell myself that events, people's behaviors, and my own attitudes "should" be the way I expected them to be and not as they really are.
EXAMPLES: "I should have been a better mother". "He should have married Ann instead of Mary". "I shouldn't have made so many mistakes."

My example:………

**Frequency:**<br>**Intensity:**	**No** (It did not occur)	**Occasional (**1-2 days during the past week)	**Much of the time (**3-5 days during the past week)	**Almost all of the time (**6-7 days during the past week)
**I believed it...**	0			
A little (Up to 30%)		1	2	3
Much (31% to 70%)		2	3	4
Very much (More than 70%)		3	4	5

12. **Jumping to conclusions (also called arbitrary inference):** I draw conclusions (negative or positive) from little or no confirmatory evidence.
EXAMPLES: "As soon as I saw him I knew he would do a lousy work." "He looked at me in a way that I immediately knew he was responsible for the accident."

My example:………

**Frequency:**<br>**Intensity:**	**No** (It did not occur)	**Occasional (**1-2 days during the past week)	**Much of the time (**3-5 days during the past week)	**Almost all of the time (**6-7 days during the past week)
**I believed it...**	0			
A little (Up to 30%)		1	2	3
Much (31% to 70%)		2	3	4
Very much (More than 70%)		3	4	5

13. **Blaming (others or oneself):** I direct my attention to others as sources of my negative feelings and experiences, failing to consider my own responsibility; or, conversely, I take responsibility for others' behaviors and attitudes.
EXAMPLES: 'My parents are the only ones to blame for my unhappiness." "It is my fault that my son married a selfish and uncaring person."

My example:………

**Frequency:**<br>**Intensity:**	**No** (It did not occur)	**Occasional (**1-2 days during the past week)	**Much of the time (**3-5 days during the past week)	**Almost all of the time (**6-7 days during the past week)
**I believed it...**	0			
A little (Up to 30%)		1	2	3
Much (31% to 70%)		2	3	4
Very much (More than 70%)		3	4	5

14. **What if?:** I keep asking myself questions such as "what if something happens?"
EXAMPLES: "What if my car crashes?" "What if I have a heart attack?" "What if my husband leaves me?"

My example:………

Frequency:<br>**Intensity:**	**No** (It did not occur)	**Occasional (**1-2 days during the past week)	**Much of the time (**3-5 days during the past week)	**Almost all of the time (**6-7 days during the past week)
**I believed it...**	0			
A little (Up to 30%)		1	2	3
Much (31% to 70%)		2	3	4
Very much (More than 70%)		3	4	5

15. **Unfair comparisons:** I compare myself with others who seem to do better than I do and place myself in a disadvantageous position.
EXAMPLES: "My father always preferred my elder brother because he is much smarter than I am." "I can't stand she is more successful than I am."

My example:………

Frequency:<br>**Intensity:**	**No** (It did not occur)	**Occasional (**1-2 days during the past week)	**Much of the time (**3-5 days during the past week)	**Almost all of the time (**6-7 days during the past week)
**I believed it...**	0			
A little (Up to 30%)		1	2	3
Much (31% to 70%)		2	3	4
Very much (More than 70%)		3	4	5

Session 21: Unhelpful thinking styles (continued)
(Stage 2: Core interventions)

Material to be distributed at this session

- Handout 3: *Bi-weekly monitoring worksheet*
- Two copies of Handout 17: *Cognitive distortions questionnaire (CD-Quest)*

Check Homework

- Review recording / self-monitoring of participants' eating behaviour and physical activity (the therapist reminds participants of the importance of having only 2s and 3s recorded in the Hunger and Satiety columns of their diary, of consuming fruit and vegetables every day in accordance with the prevailing national guidelines, and of the importance of gradually attaining and maintain physical activity levels of approximately 8,000 to 12,000 steps per day or its equivalent).
- Review participants' responses to the *Cognitive distortions questionnaire (CD-Quest*, Handout 17), and discuss their experiences of trying to identify any distorted or dysfunctional thoughts they may have.

Bi-weekly monitoring of participants' progress with behavioral weight loss therapy

In this activity, the therapist's aims are the following:
- Encourage participants to improve the quality of their eating behavior recording.
- Assess participants' progress with the program.
- Identify any difficulties or barriers to change and assist with strategies to resolve these.

The *bi-weekly monitoring worksheet* (Handout 3) is again introduced to participants and the monitoring is done in session. The therapist should remind participants to transfer their data from the *Bi-weekly monitoring worksheet* to their *HAPIFED summary sheet* (Handout 2), and any emerging patterns should be discussed in order to assist participants in achieving the goals of eating within hunger and satiety levels of ±2 and ±3, achieving adequate intake of fruit and vegetables, and achieving physical activity levels commensurate with long-term successful weight loss (8,000 to 12,000 steps per day or its equivalent).

Unhelpful thinking styles and disordered eating behaviors (continued)

In this activity, the therapist's aim is to help participants to cope with situations where they experience dysfunctional thoughts that trigger disordered eating.

The psychopathology of eating disorders may be viewed as a "mindset" (1). The eating disorders mindset is formed by dysfunctional beliefs related to body image and eating, such as the overvaluation of the importance of one's body shape, and dichotomous thinking about healthy eating (1). Because of that, it is important that participants identify circumstances that trigger this eating disorders mindset, and that they know what to do in these situations. To aid this process, the therapist can invite participants to write as much detail as they can remember about the following:

1. The last time you remember engaging in disordered eating behaviors (e.g. binge eating, not eating despite hunger, purging, excessive exercise, eating when not hungry). When / where was it? What did you do?
2. What may have influenced your engagement with these behaviors (e.g. an adverse event, unpleasant emotional states)?
3. What were your thoughts at that moment? Are there other ways of thinking about that situation that could have been more helpful?
4. What could you have done differently in that situation, in order to avoid engaging in disordered eating?

In order to help participants with the activity, the therapist can state that there are circumstances that are known to trigger the eating disorders mindset, such as situations that highlight weight and shape (e.g. going to the beach in summer and comparing one's body to that of others), as well as adverse events that trigger changes in mood (1). The therapist can also help participants with ideas that they could try when experiencing the eating disorders mindset (e.g. going for a walk, arranging to meet friends). It is recommended that the outcomes of this activity be discussed during the session with the group, as participants may gain insight from others' experiences with what triggers the disordered eating mindset, and how to deal with it in healthy ways.

Exercise on cognitive distortions

Participants are invited by the therapist to again answer the *Cognitive distortions questionnaire (CD-Quest*, Handout 17) in session. The aim of this activity is to help participants to notice how much and what type of cognitive distortions they may be engaging in, thereby helping them to be more aware of any unhelpful thinking styles that may contribute to their disordered eating. In this activity, it is recommended that the therapist instructs the participants to answer the *Cognitive distortions questionnaire* focusing on potential cognitive distortions related to body image or eating.

Homework

- Participants continue recording / self-monitoring their eating behavior and physical activity.
    - Remind participants to bring their *HAPIFED Diary* (Handout 1) *and HAPIFED summary sheet* (Handout 2) to the next session.
- Participants are asked to answer the *Cognitive distortions questionnaire (CD-Quest*, Handout 17) again at home, using a fresh copy of Handout 17, in order to help increase their awareness of distorted or dysfunctional thinking styles.
    - Remind participants to bring their *Cognitive distortions questionnaire (CD-Quest)* to the next session.

Session 22: Getting social support
(Stage 2: Core interventions)

Material to be distributed at this session

- Handout 20: *Getting social support*

Check homework

- Review recording / self-monitoring of participants' eating behaviour and physical activity (the therapist reminds participants of the importance of having only 2s and 3s recorded in the Hunger and Satiety columns of their diary, of consuming fruit and vegetables every day in accordance with the prevailing national guidelines, and of the importance of gradually attaining and maintain physical activity levels of approximately 8,000 to 12,000 steps per day or its equivalent).
- Review participants' responses to the *Cognitive distortions questionnaire (CD-Quest*, Handout 19) and discuss their experiences of trying to identify any distorted or dysfunctional thoughts they may have. This week, participants are encouraged to think about the influence of any cognitive distortions they may have on their eating behavior. As part of this, the therapist can ask participants to brainstorm examples as a group.

Getting social support

In this activity, the therapist is to aid participants in strengthening their social support from people or groups of people that could potentially help them to improve their eating behavior. The therapist can introduce this activity by asking participants if they remember any situation(s) where a family member, friend or group of people supported them in achieving an important goal that they were struggling to achieve. The aim of the therapist in this introductory conversation should be to emphasize the importance of connecting with people that are supportive in order to overcome difficulties. Next, the therapist can ask participants if they think that participating in activities with supportive people could also be helpful in order to help them improve their behavior in regards to eating and exercising. Finally, it is advised that the therapist ask participants to brainstorm ideas on how to get social support that could potentially facilitate healthy eating and exercise habits. The therapists can provide some examples of activities that can potentially promote appropriate social support to maintain healthy eating and healthy exercise habits (e.g. to plant vegetables in a community garden, join a walking or yoga group, join an online group that is supportive of a healthy lifestyle etc). It is recommended that the therapist provide Handout 18 (*Getting social support*) to participants before starting the brainstorming group discussion, so that participants can write on the handout any ideas that appeal to them about getting appropriate social support.

Homework

- Participants continue recording / self-monitoring their eating behavior and physical activity.
    - Remind participants to bring their *HAPIFED Diary* (Handout 1) *and HAPIFED summary sheet* (Handout 2) to the next session.
- Participants are asked to review / complete Handout 18 (*Getting social support*), and to bring this handout to the next session.

30 Group Session Program Manual © da Luz, Swinbourne, Hay, Touyz, Palavras, Claudino, Sainsbury 2017

Handout 18: Getting social support

What activities could you participate in that would potentially provide social support to help you attain or maintain healthy eating habits and healthy exercise habits? Please write as many activities that you can think of (even if they don't seem do-able at this time).

1. ………………………………………………………………………………………
2. ………………………………………………………………………………………
3. ………………………………………………………………………………………
4. ………………………………………………………………………………………
5. ………………………………………………………………………………………
6. ………………………………………………………………………………………
7. ………………………………………………………………………………………
8. ………………………………………………………………………………………
9. ………………………………………………………………………………………
10. ………………………………………………………………………………………

From the options that you listed above, is there any activity that you could start in the near future? If yes, what are the steps that you would need to take in order to engage in this activity?

……

……

……

……

……

What could be the advantages of starting this activity?

……

……

……

……

……

Session 23: Positive self-evaluation
(Stage 2: Core interventions)

Material to be distributed at this session

- Handout 1: *HAPIFED formulation of disordered eating and weight gain*
- Handout 3: *Bi-weekly monitoring worksheet*
- Handout 19: *Activities that promote positive self-evaluation*

Check Homework

- Review recording / self-monitoring of participants' eating behaviour and physical activity (the therapist reminds participants of the importance of having only 2s and 3s recorded in the Hunger and Satiety columns of their diary, of consuming fruit and vegetables every day in accordance with the prevailing national guidelines, and of the importance of gradually attaining and maintain physical activity levels of approximately 8,000 to 12,000 steps per day or its equivalent).
- Review participants' ideas for getting appropriate social support using Handout 18 (*Getting social support*)

Bi-weekly monitoring of participants' progress with behavioral weight loss therapy

At this activity, the therapist aims are the following:
- Encourage participants to improve the quality of their eating behavior recording.
- Assess participants' progress with the program.
- Identify any difficulties or barriers to change and assist with strategies to resolve these.

The *Bi-weekly monitoring worksheet* (Handout 3) should be used and the monitoring is done in session. The therapist should remind participants to transfer their data from the *Bi-weekly monitoring worksheet* to their *HAPIFED summary sheet* (Handout 2), and any emerging patterns should be discussed in order to assist participants in achieving the goals of eating within hunger and satiety levels of ±2 and ±3, achieving adequate intake of fruit and vegetables, and achieving physical activity levels commensurate with long-term successful weight loss (8,000 to 12,000 steps per day or its equivalent).

30 Group Session Program Manual © da Luz, Swinbourne, Hay, Touyz, Palavras, Claudino, Sainsbury 2017

Promoting positive self-evaluation through activities not related to self-evaluation based on weight, shape or eating

Individuals with eating disorders tend to evaluate themselves mainly (and negatively) with regards to their body image, notably their weight or shape, as well as the type and quantity of foods and beverages they consume, and other areas of their lives are often marginalized (1). As an introduction to this activity, it is recommended that the therapist explain that it is very common for people to feel long-term dissatisfaction with their weight or shape, but that self-evaluation that is excessively focused on weight or shape is unhealthy and can contribute to the maintenance of disordered eating. The therapist can use the *HAPIFED formulation of disordered eating and weight gain* (Handout 1) to discuss this point with participants.

Next, the therapist can encourage participants' engagement in activities that they enjoy and that are likely to positively affect their self-evaluation, independently of body image concerns around weight or shape. These activities should be of the type that participants are likely to feel valued from due to positive characteristics that they have or admirable behaviors that they engage in. It is important that these positive characteristics or behaviors are not related to a "perfect" ideal of their body or to "perfect" eating, because these are impossible targets that contribute to frustration and disordered eating. Examples of appropriate types of activities are playing musical instruments, painting, joining a theatre group, writing a book, volunteering to help others in need, meeting friends, etc. After introducing this activity based on the above information, it is recommended that the therapist ask participants to work on Handout 19 (*Activities that promote positive self-evaluation*). After participants have completed Handout 19, it is recommended that the therapist ask participants to share their ideas with group (if the feel comfortable doing so), because this can aid participants that may find it difficult to think about helpful activities that they can engage in.

Homework

- Participants continue recording / self-monitoring their eating behavior and physical activity.
  - Remind participants to bring their *HAPIFED Diary* (Handout 1) *and HAPIFED summary sheet* (Handout 2) to the next session.
- Participants are asked to review Handout 19 (*Activities that promote positive self-evaluation*) at home, add further information if necessary, and bring this handout to the next session.
- Participants are invited to engage in activities that promote positive self-evaluation and are not related to self-evaluation based on weight, shape or eating (according to what they wrote in Handout 19). Participants are reminded to bring this handout to the next session.

Handout 19: Activities that promote positive self-evaluation

What activities do you enjoy (or have ever enjoyed or would potentially enjoy) that are likely to make you feel good about yourself independently of body image concerns around weight or shape? Please list as many activities of this type that you can think of, even if they don't seem do-able at this moment.

1. ………………………………………………………………………………………
2. ………………………………………………………………………………………
3. ………………………………………………………………………………………
4. ………………………………………………………………………………………
5. ………………………………………………………………………………………
6. ………………………………………………………………………………………
7. ………………………………………………………………………………………
8. ………………………………………………………………………………………
9. ………………………………………………………………………………………
10. ………………………………………………………………………………………

From the options that you listed above, is there any activity that you could start in the near future? If yes, what are the steps that you would need to take in order to engage in this activity?

……………………………………………………………………………………………………

……………………………………………………………………………………………………

……………………………………………………………………………………………………

……………………………………………………………………………………………………

……………………………………………………………………………………………………

What could be the advantages of starting this activity?

……………………………………………………………………………………………………

……………………………………………………………………………………………………

……………………………………………………………………………………………………

……………………………………………………………………………………………………

……………………………………………………………………………………………………

Session 24: Positive self-evaluation (continued)
(Stage 2: Core interventions)

Material to be distributed at this session

- Handout 5: *Proactive problem solving sheet*
- Handout 19: *Activities that promote positive self-evaluation*

Check Homework

- Review recording / self-monitoring of participants' eating behaviour and physical activity (the therapist reminds participants of the importance of having only 2s and 3s recorded in the Hunger and Satiety columns of their diary, of consuming fruit and vegetables every day in accordance with the prevailing national guidelines, and of the importance of gradually attaining and maintain physical activity levels of approximately 8,000 to 12,000 steps per day or its equivalent).
- Check if participants reviewed Handout 19 (*Activities that promote positive self-evaluation*), and how this may influence their plans with regards to activities that promote positive self-evaluation.
- Review participants' experiences of engaging in activities that promote positive self-evaluation and are not related to self-evaluation based on weight, shape or eating.

Promoting positive self-evaluation through activities not related to self-evaluation based on weight, shape, or eating (continued)

In this activity, the therapist is to continue encouraging participants to engage in activities that make them feel good about themselves independently of body image concerns around weight or shape. It is recommended that the therapist checks participants' progress with engagement or intention to engage in this type of activity. It is advised that the therapist ask participants who face barriers with engaging in this type of activity to work on proactive problem solving using Handout 5. Some participants may not have been able to come up with an activity of this type that they could engage in. If there are any participants in this situation, it is advised that the therapist ask them to review Handout 19 (*Activities that promote positive self-evaluation*) in session. In this case, the therapist can help them with a few suggestions and/or ask other participants to share types of activities that could potentially work for them. Some participants may not seem motivated to engage in this type of activity. For participants in this situation, the therapist can ask them to write a list of disadvantages and advantages of engaging in one of these activities. In this case, it is recommended that the therapist help participants realize the benefits of engaging in this type of activity.

Homework

- Participants continue recording / self-monitoring their eating behavior and physical activity.
    - Remind participants to bring their *HAPIFED Diary* (Handout 1) *and HAPIFED summary sheet* (Handout 2) to the next session.
- Participants are invited to engage in activities that promote positive self-evaluation and are not related to self-evaluation based on weight, shape or eating.

Session 25: Nutritional education (final review)
(Stage 2: Core interventions)

Material to be distributed at this session

- Nutritional education material
- Handout 3: *Bi-weekly monitoring worksheet*

Check Homework

- Review recording / self-monitoring of participants' eating behaviour and physical activity (the therapist reminds participants of the importance of having only 2s and 3s recorded in the Hunger and Satiety columns of their diary, of consuming fruit and vegetables every day in accordance with the prevailing national guidelines, and of the importance of gradually attaining and maintain physical activity levels of approximately 8,000 to 12,000 steps per day or its equivalent).
- Review participants' experiences of engaging in activities that promote positive self-evaluation and are not related to self-evaluation based on weight, shape or eating.

Bi-weekly monitoring of participants' progress with behavioral weight loss therapy

In this activity, the therapist's aims are the following:

- Encourage participants to improve the quality of their eating behavior recording.
- Assess participants' progress with the program.
- Identify any difficulties or barriers to change and assist with strategies to resolve these.

The *Bi-weekly monitoring worksheet* (Handout 3) is again introduced to participants and the monitoring is done in session. The therapist should remind participants to transfer their data from the *Bi-weekly monitoring worksheet* to their *HAPIFED summary sheet* (Handout 2), and any emerging patterns should be discussed in order to assist participants in achieving the goals of eating within hunger and satiety levels of ±2 and ±3, achieving adequate intake of fruit and vegetables, and achieving physical activity levels commensurate with long-term successful weight loss (8,000 to 12,000 steps per day or its equivalent).

Nutritional education with dietitian (final review)

Suggested topics:

- Review of key points from Session 4.
- Review of changes of participants' eating behavior.
- Address participants' questions.

At the end of this session, the therapist can explain that there is not a set topic for the next session, meaning that participants can decide what topic(s) they would like covered. The therapist can remind participants of the topics covered in the previous sessions and use prompts such as "is there any topic that you would like to see covered in more detail?", "Is there any topic that you have questions about?", "Is there any other topic that wasn't discussed and you would like to talk about?", "Are there any barriers that we could discuss in more detail?" The outcome of this discussion can then be used to set the agenda for the next session, which will be agreed upon by the participants and the therapist prior to completion of the session.

Homework

- Participants continue recording / self-monitoring their eating behavior and physical activity.
    - Remind participants to bring their *HAPIFED Diary* (Handout 1) *and HAPIFED summary sheet* (Handout 2) to the next session.

30 Group Session Program Manual © da Luz, Swinbourne, Hay, Touyz, Palavras, Claudino, Sainsbury 2017

Session 26: Open session
(Stage 2: Core interventions)

There is no new material to be distributed at this session

Check Homework

- Review recording / self-monitoring of participants' eating behaviour and physical activity (the therapist reminds participants of the importance of having only 2s and 3s recorded in the Hunger and Satiety columns of their diary, of consuming fruit and vegetables every day in accordance with the prevailing national guidelines, and of the importance of gradually attaining and maintain physical activity levels of approximately 8,000 to 12,000 steps per day or its equivalent).

Open session

The topic that was set in Session 25 is explored in this session.

Homework

- Participants continue recording / self-monitoring their eating behavior and physical activity.
  - Remind participants to bring their *HAPIFED Diary* (Handout 1) *and HAPIFED summary sheet* (Handout 2) to the next session.

Stage 3: Relapse prevention

Session 27: Relapse prevention (Part 1)
(Stage 3: Relapse prevention)

Material to be distributed at this session

- Handout 3: *Bi-weekly monitoring worksheet*
- Handout 20: *My plan going forwards*

Check Homework

- Review recording / self-monitoring of participants' eating behaviour and physical activity (the therapist reminds participants of the importance of having only 2s and 3s recorded in the Hunger and Satiety columns of their diary, of consuming fruit and vegetables every day in accordance with the prevailing national guidelines, and of the importance of gradually attaining and maintain physical activity levels of approximately 8,000 to 12,000 steps per day or its equivalent).

Bi-weekly monitoring of participants' progress with behavioral weight loss therapy

In this activity, the therapist's aims are the following:

- Encourage participants to improve the quality of their eating behavior recording.
- Assess participants' progress with the program.
- Identify any difficulties or barriers to change and assist with strategies to resolve these.

The *Bi-weekly monitoring worksheet* (Handout 3) is again introduced to participants and the monitoring is done in session. The therapist should remind participants to transfer their data from the *Bi-weekly monitoring worksheet* to their *HAPIFED summary sheet* (Handout 2), and any emerging patterns should be discussed in order to assist participants in achieving the goals of eating within hunger and satiety levels of ±2 and ±3, achieving adequate intake of fruit and vegetables, and achieving physical activity levels commensurate with long-term successful weight loss (8,000 to 12,000 steps per day or its equivalent).

30 Group Session Program Manual © da Luz, Swinbourne, Hay, Touyz, Palavras, Claudino, Sainsbury 2017

My plan going forwards

As an introduction to this activity, the therapist is to address any concerns that participants may have about ending the HAPIFED Program. It is recommended that the therapist emphasize that participants learned skills throughout the program that they can use to improve their eating behavior or maintain the improvements that they achieved even after the program has finished. The aim of the therapist is to improve participants' self-efficacy to overcome disordered eating. Because of that, it is important to inform participants that setbacks may occur, and that it is not helpful to interpret these setbacks as a complete "relapse" (back to the stage that they were before the program) (1). Instead, these occasions should be interpreted as a "lapse", which means a temporary moment of difficulty that can be overcome using the skills that were learned during the program.

Next, the therapist's aim is to help prepare participants for the end of the HAPIFED Program and promote relapse prevention. The therapist can ask participants to answer the questions in Handout 20, entitled *My plan going forwards*. After participants have answered all of the questions, they can be invited to share their plan with the group (if they feel comfortable to do so), because their ideas can be useful to other participants. If there is not enough time to complete this activity in session, participants are asked to finish it at home.

Homework

- Participants continue recording / self-monitoring their eating behavior and physical activity.
    - Remind participants to bring their *HAPIFED Diary* (Handout 1) *and HAPIFED summary sheet* (Handout 2) to the next session.
- Participants are asked to finish answering or review their answers to *My plan going forwards* (Handout 20), and bring this handout to the next session.

Handout 20: My plan going forwards

1. **What strategies and procedures do I want to continue?**

2. **What are my expectations? Are these realistic?**

3. **What is the difference between a lapse and a relapse?**

4. **What are my high-risk times?**

5. **What is my plan for dealing with setbacks?**

Session 28: Relapse prevention (Part 2)

(Stage 3: Relapse prevention)

<u>There is no new material to be distributed at this session</u>

<u>Check Homework</u>

- Review recording / self-monitoring of participants' eating behaviour and physical activity (the therapist reminds participants of the importance of having only 2s and 3s recorded in the Hunger and Satiety columns of their diary, of consuming fruit and vegetables every day in accordance with the prevailing national guidelines, and of the importance of gradually attaining and maintain physical activity levels of approximately 8,000 to 12,000 steps per day or its equivalent).
- Review and discuss participants' answers to *My plan going forwards* (Handout 20). If participants have not completed this plan, it is recommended that they do this in session.

<u>My plan going forwards</u> (continued)

In this activity, the therapist is to invite participants to explore in detail the possible scenarios that may present as high risk for a lapse. In doing this, it may be useful to ask participants to think back to previous lapses and identify the reasons why they lapsed. Ultimately, the aim of this activity is for participants to find solutions to scenarios that may contribute to disordered eating. It is recommended that the therapist ask participants to generate a discussion whereby each participant identifies an individual high-risk situation (either from past experiences or anticipated), and the group brainstorms possible solutions. It is important that the therapist emphasizes that the ideas brainstormed by the group in the discussion are only ideas to consider, and that no participant is telling another what to do. Next, the therapist leads a debriefing about this activity and encourages participants to add any new ideas to their own *plan going forwards* (Handout 20).

<u>Homework</u>

- Participants continue recording / self-monitoring their eating behavior and physical activity.
  - Remind participants to bring their *HAPIFED Diary* (Handout 1) *and HAPIFED summary sheet* (Handout 2) to the next session.
- Review Handout 20, entitled *My plan going forwards*, and bring this to the next session.

Session 29: Relapse prevention (Part 3)

(Stage 3: Relapse prevention)

Material to be distributed at this session

- Handout 3: *Bi-weekly monitoring worksheet*
- Handout 21: *A letter to myself about my long-term maintenance plan*

Check Homework

- Review recording / self-monitoring of participants' eating behaviour and physical activity (the therapist reminds participants of the importance of having only 2s and 3s recorded in the Hunger and Satiety columns of their diary, of consuming fruit and vegetables every day in accordance with the prevailing national guidelines, and of the importance of gradually attaining and maintain physical activity levels of approximately 8,000 to 12,000 steps per day or its equivalent).
- Review and discuss participants' responses to Handout 20, entitled *My plan going forwards*.

Bi-weekly monitoring of participants' progress with behavioral weight loss therapy

In this activity, the therapist's aims are the following:

- Encourage participants to improve the quality of their eating behavior recording.
- Assess participants' progress with the program.
- Identify any difficulties or barriers to change and assist with strategies to resolve these.

The *Bi-weekly monitoring worksheet* (Handout 3) should be used and the monitoring is done in session. The therapist should remind participants to transfer their data from the *Bi-weekly monitoring worksheet* to their *HAPIFED summary sheet* (Handout 2), and any emerging patterns should be discussed in order to assist participants in achieving the goals of eating within hunger and satiety levels of ±2 and ±3, achieving adequate intake of fruit and vegetables, and achieving physical activity levels commensurate with long-term successful weight loss (8,000 to 12,000 steps per day or its equivalent).

A letter to myself about my long-term maintenance plan

In this activity, the therapist invites participants to write a letter to themselves about their long-term maintenance plan (Handout 21). It is recommended that the therapist encourage participants to write a frank and reassuring letter that they can read in the future, in moments when they feel that they may be losing motivation to maintain healthy eating behaviors. For example, the letter can include reminders about what they can do if they feel off track or are losing motivation to maintain change. It is important that the therapist inform participants that there is no need to share the content of their letter with the other participants, because the letter may be very personal.

Homework

- Participants continue recording / self-monitoring their eating behavior and physical activity.
    - Remind participants to bring their *HAPIFED Diary* (Handout 1) *and HAPIFED summary sheet* (Handout 2) to the next session.
- Participants review their letter to themselves (Handout 21), and add information or rewrite it if they think it can be improved, and bring this to the next session.

30 Group Session Program Manual © da Luz, Swinbourne, Hay, Touyz, Palavras, Claudino, Sainsbury 2017

Handout 21: A letter to myself about my long-term maintenance plan

Please write a frank and encouraging letter to yourself that you can read and re-read, particularly in times of difficulties with eating. You may like to include some of the following points in your letter:

• **My goals**<br>• **Strategies I want to continue**<br>• **How will I review my progress?**<br>• **How will I minimize the risk of setbacks?**<br>• **What might increase the risk of a setback?**<br>• **What are the early warning signs of a lapse?**	• **How can I deal with triggers and setbacks?**<br>• **Am I eating according to my hunger and satiety?**<br>• **Am I eating the nationally recommended amounts of fruits and vegetables?**<br>• **Am I engaging in regular physical activity?**

Session 30: Relapse prevention (Part 4)
(Stage 3: Relapse prevention)

There is no new material to be distributed at this session

Check homework

- Review recording / self-monitoring of participants' eating behaviour and physical activity (the therapist reminds participants of the importance of having only 2s and 3s recorded in the Hunger and Satiety columns of their diary, of consuming fruit and vegetables every day in accordance with the prevailing national guidelines, and of the importance of gradually attaining and maintain physical activity levels of approximately 8,000 to 12,000 steps per day or its equivalent).
- Check that participants reviewed their letters to themselves (Handout 21), and briefly discuss strategies that the participants' want to continue.

This session includes two activities that are explained below.

Reflection on the outcomes of the therapy

In this activity, the therapist is to remind participants that the aims of the HAPIFED Program were:

1. To promote strategies for healthy weight management (eating varied and nutritious foods according to physical hunger, combined with healthy physical activity).
2. To reduce binge eating, self-induced vomiting and other eating disorder behaviors.
3. To promote healthy self-evaluation (not dominated by concerns related to food, eating, weight or shape).

The therapist is to explain that it is possible that some participants may have not completely reached these goals by the end of the program, but that any improvements with regards to the above-mentioned program aims should be acknowledged. It is recommended that the therapist also explain that the timing of change is very individual; some participants may be on the way to making changes and will need more time and practice in order to start improving their eating behavior. It is important to emphasize that this is the start of a journey of improved mental and physical wellbeing.

Note: some participants may face other circumstances that negatively affect their eating behavior (e.g. mental health problems, financial difficulties, medical problems) and may require additional support. It is important that the therapist provides referral to other services (e.g. mental health services, social support services, GPs) for participants as necessary. Referral to additional eating disorder programs or weight loss programs should also be provided to participants if appropriate.

Achievements, learning and encouragement

In this activity, the therapist is to finish the HAPIFED Program with a positive attitude towards overcoming disordered eating. It is recommended that the therapist ask participants to share the following with the group:

1. Activities that were useful to them in order to improve their problems with eating.
2. Something that they learned or realized during the program that helped them to improve their eating behavior.
3. A positive and encouraging message to the other members of the group.

It is recommended that the therapist close the HAPIFED Program with a positive affirmation for the group and individual participants, thanking all the participants for their contributions, and describing an individual example of each contribution.

30 Group Session Program Manual © da Luz, Swinbourne, Hay, Touyz, Palavras, Claudino, Sainsbury 2017

List of handouts

Number	Title	Page
1	HAPIFED Diary	10
2	HAPIFED summary sheet	18
3	Bi-weekly monitoring worksheet	25
4	Case vignettes	27
5	Proactive problem solving sheet	31
6	Healthy and unhealthy exercise	35
7	Reviewing a situation that triggered disordered eating	49
8	Action plan	52
9	Interpersonal conflicts	55
10	Aim: Focus on positive aspects of your body	60
11	Identifying my good points	63
12	Self-nurturing activities	67
13	Fifteen steps to a more confident you	73
14	Slow breathing	76
15	Progressive muscle relaxation	79
16	Personalizing the HAPIFED formulation of loss of control over eating and weight gain	82
17	Cognitive distortions questionnaire (CD-Quest)	86
18	Getting social support	95
19	Activities that promote positive self-evaluation	98
20	My plan going forwards	107
21	A letter to myself about my long-term maintenance plan	111

References

1. Fairburn C. Cognitive behavior therapy and eating disorders. New York: Guilford; 2008.
2. Sainsbury-Salis A. The don't go hungry diet. Australia: Bantam; 2007.
3. Sainsbury-Salis A. Don't go hungry for life. Australia: Bantam; 2011.
4. Touyz S, Polivy J, Hay P. Eating disorders: advances in psychotherapy – Evidence-Based Practice 2008.
5. Brownell KD. The LEARN program for weight management. Dallas, TX: American Health; 2000.
6. American Psychiatric Association. Diagnostic and statistical manual of mental disorders: fifth edition. 5th ed2013.
7. Sheehan DV, Lecrubier Y, Sheehan KH, Amorim P, Janavs J, Weiller E, et al. The Mini-International Neuropsychiatric Interview (M.I.N.I.): the development and validation of a structured diagnostic psychiatric interview for DSM-IV and ICD-10. J Clin Psychiatry. 1998;59 Suppl 20:22-33;quiz 4-57.
8. Cooper P. Overcoming bulimia nervosa and binge-eating: A self-help guide using cognitive behavioral techniques. London: Robinson; 2010.
9. National Health and Medical Research Council. Australian Dietary Guidelines: Canberra: National Health and Medical Research Council; 2013.
10. Williams M, Teasdale J, Segal Z, Kabat-Zinn J. The mindful way through depression: freeing yourself from chronic unhapiness. New York: Guilford Press; 2007.
11. Bedell JR, Lennox SS. Handbook for communication and problem-solving skills training: a cognitive-behavioral approach: Wiley; 1997.
12. Noordenbos G. Recovery from Eating Disorders: A Guide for Clinicians and Their Clients. Oxford: Wiley-Blackwell; 2013. p. 81-2.
13. Hilbert A, Tuschen-Caffier B. Maintenance of binge eating through negative mood: A naturalistic comparison of binge eating disorder and bulimia nervosa. International Journal of Eating Disorders. 2007;40(6):521-30.
14. Lo Sauro C, Ravaldi C, Cabras PL, Faravelli C, Ricca V. Stress, Hypothalamic-Pituitary-Adrenal Axis and Eating Disorders. Neuropsychobiology. 2008;57(3):95-115.
15. Steiger H, Thaler L. Eating disorders, gene-environment interactions and the epigenome: Roles of stress exposures and nutritional status. Physiology & behavior. 2016;162:181-5.
16. De Oliveira IR. Trial-Based Cognitive Therapy: A Manual for Clinicians: Taylor & Francis; 2014.
